# Identification of biological components for sialolith formation organized in circular multi-layers

**DOI:** 10.1038/s41598-023-37462-w

**Published:** 2023-07-28

**Authors:** Buyanbileg Sodnom-Ish, Mi Young Eo, Yun Ju Cho, Mi Hyun Seo, Hyeong-Cheol Yang, Min-Keun Kim, Hoon Myoung, Suk Keun Lee, Soung Min Kim

**Affiliations:** 1 Department of Oral and Maxillofacial Surgery, School of Dentistry, Dental Research Institute, Seoul National University, Seoul, Korea; 2 Department of Dental Biomaterials Science, School of Dentistry, Dental Research Institute, Seoul National University, Seoul, Korea; 3 Department of Oral and Maxillofacial Surgery, College of Dentistry, Gangneung-Wonju National University, Gangneung, Korea; 4 Department of Oral Pathology, College of Dentistry, Gangneung-Wonju National University, Gangneung, Korea

**Keywords:** Diseases, Pathogenesis

## Abstract

According to the previous studies of sialolithiasis reported so far, this study is aimed to identify the biological components of sialolith, which show different ultrastructures and chemical compositions from other stones, cholelith and urolith. Twenty-two specimens obtained from 20 patients were examined histologically, and analyzed with micro-CT, scanning electron microscopy (SEM), energy dispersive X-ray spectroscopy (EDS), and transmission electron microscopy (TEM). All sialoliths (n = 22) observed in this study showed a central nidus, which was filled with organoid matrix admixed with exosome vesicles, loose calcium apatite crystals, and many bacteria. The micro-CT and SEM observation clearly defined a single or multiple central nidus(es) encircled by highly calcified compact zone. The circular compact zone showed a band-like calcification, about 1–3 mm in thickness, and usually located between the central nidus and the peripheral multilayer zone. But some sialoliths (n = 5) showed severe erosion of compact zone by expanding multilayered zone depending on the level of calcification and inflammation in sialolith. By observing TEM images, many exosome vesicles and degraded cytoplasmic organelles were found in the central nidus, and some epithelial cells were also found in the calcified matrix of peripheral multilayer zone. Particularly, EDS analysis indicated the highest Ca/P ratio in the intermediate compact zone (1.77), and followed by the central nidus area (1.39) and the peripheral multilayer zone (0.87). Taken together, these data suggest that the central nidus containing many inflammatory exosomes and degraded cytoplasmic organelles has a potential to induce a band-like calcification of compact zone, and followed by the additional multilayer deposition of exfoliated salivary epithelial cells as well as salivary materials. Thereby, the calcium apatite-based sialolith is gradually growing in its volume size, and eventually obstructs the salivary flow and provides a site for the bacterial infection.

## Introduction

Sialolithiasis is a common disease of salivary glands characterized by the obstruction of the salivary secretion by a calculus. The incidence of sialolithiasis is reported to be 0.115%, 23 cases were found among 20,000 patients during 18 years in a stomatologic institute by de Temiño and Villar y Pérez de los Ríos^1–3^. The diagnosis is based on the medical history, clinical findings, physical examination, and diagnostic imaging including sialography, ultrasonography, computed tomography (CT), and magnetic resonance imaging^4^. Ultrasound and CT scans are the methods of choice for diagnosis.

Preventative measures such as good salivary secretory activity and a diet with a reasonable content of phytate have been summarized recently based on clinical and experimental research^5^. The treatment protocol for sialolithiasis is based on the anatomical location, size, and accessibility of the sialoliths depending on the type of salivary gland^6^. The recent advances in sialolith treatment show that shock-waves are best suited for the fragmentation of large sialoliths, whereas laser ablation can be useful for destroying small sialoliths or fragments generated by other lithotripsy techniques^7^. However, the efficacy of the lithotripsy method for sialolithiasis is relatively lower than that of the treatment for renal stones, which primarily consist of calcium stones^7,8^. The underlying reason is that ultrasonic waves target specifically mineralized material leaving the organic material largely unaffected^7^. Intraductal shock-wave lithotripsy is effective but not the fastest method of treatment for difficult sialolithiasis, especially in the parotid gland which may require increased efforts using combination treatment^6,9^. In a retrospective study, sialolithiasis was found to be the most frequent pathology requiring the extirpation of the submandibular gland, accounting for 73.5% of cases. This was followed by benign tumors at 18.5% and malignant tumors at 8%^10^. The surgical resection of salivary glands is associated with several unfavorable outcomes, including cosmetic aspects, reduced saliva production, collateral damage to the lingual and facial nerves, and the potential development of Frey’s syndrome^6^. Therefore, there is a great demand for research and development of a reliable treatment method for sialolithiasis without morbidity and sequelae to the patients.

Among human calcium phosphate calculi or stones, there exist dental calculus, sialoliths, uroliths, and nephroliths. In addition, rhinoliths, antroliths, tonsilloliths, pancreatic calculus, uterine stones, gallstones, and other stones are also known^11^. Kidney stones have been formally studied since 1802. There is an extensive study on the pathophysiology, microstructure, chemistry, microbiome composition, prevention, and treatment of kidney stone formation^12–18^. Although numerous studies have been conducted on kidney stones and urinary tract stones, the exact pathogenesis of sialoliths is still not fully understood.

Many theories have been proposed on sialolith formation, which include (1) the organic core theory (calcification of bacteria, foreign body, or desquamated epithelial cells); (2) the sialomicrolith theory (normally present in 80% of submandibular glands); and (3) the mucoepidermoid gel theory (calcification of high viscosity mucins)^2,19^.

The most comprehensive and solid observations are the occurrence of sialomicroliths, which are found in normal salivary glands of asymptomatic individuals^2^. A recent study observed that the induction of an inflammatory reaction can cause an influx of neutrophils into the salivary duct. The activation of these cells can form neutrophil extracellular traps (NETs), which are potent attractors of calcium-based crystals (hydroxyapatite, brushite, and whitlockite), and promote the pathogenesis of sialolithiasis^20^. According to the spectroscopic studies of stones by Tretiakow et al., three types of sialolith classifications were proposed: calcified, organic/lipid, and mixed^21^. Major advances in the knowledge of structure, etiology and pathogenesis of sialoliths are based on extensive clinical and experimental investigations towards the end of the last century and in this century. However, a comprehensive understanding of the nucleation and development of sialolith is still lacking.

In the present study, twenty-two sialoliths were utilized to know their micro- and ultra-structures through micro-CT, SEM, EDS, and histological and electron microscopical observation. Three main different zones of scientific interests of the cross-sectioned sialolith were investigated and localized in central nidus, intermediate circular compact, and peripheral multilayer. This methodology strategy provides a means to our understanding on finding several relevant biological components including exosomes, degraded cytoplasmic organelles, epithelial cells, bacteria, and different calcium phosphate apatite crystals; which might be important factors to explain the mechanism of sialolith formation.

## Materials and methods

### Sialolith specimen collection

We analyzed sialoliths retrieved in the Department of Oral Maxillofacial Surgery of Seoul National University School of Dentistry. The sialoliths were obtained via sialolithotomy, sialendoscopy, direct extraction, or spontaneous extrusion. Sialolithotomy was carried out with a direct linear incision over the duct to expose the sialolith taking care to preserve the lingual nerve. For sialoendoscopy, a probe was carefully inserted into the duct and expanded using a dilator. With enough access to the duct, an endoscope was inserted with saline irrigation and the sialolith was confirmed. The sialolith was then retrieved using a three-wire basket. For sialoliths located at the orifice of the duct, they were grasped with a dental pincette and removed through blunt dissection. Specimens were collected from January 2017 to July 2022 and preserved without fixation or in 10% buffered formalin, or 2.5% glutaraldehyde (GA) fixation solution as routine work for further study. The specimen was screened, and only well-preserved and intact stone samples met the criteria for further experiments. In this study we tried to employ all of the samples in each investigation technique. However due to the size and the fragility of the sialoliths not all samples were available for each investigation technique therefore, some samples were skipped, and the representative samples were presented.

Twenty-two specimens were obtained from 20 eligible patients, including 12 female patients and 8 male patients, with a mean age of 36.52 years old (ranging from 7 to 68 years old). All patients were presented with chronic sialadenitis characterized by pain and swelling in the submandibular and parotid gland region. The investigated sialoliths are marked as Si (i = 1,…,20).

### Micro-CT analysis

All specimens were subjected to high-resolution micro-CT scanning Skyscan 1273® (Bruker, Kontich, Belgium) with a 0.3 mm copper filter, source current of 136 μA, source voltage of 110 kV and resolution of 18 μm. The specimens were rotated over 360° with 0.3° steps for each X-ray image. The specimens were mounted in gauge with phosphate-buffered saline (PBS) sheath buffer, as this material is remarkably X-ray lucent. To evaluate the average attenuation, calibration rod pairs composed of epoxy resin with embedded fine CaHA powder at concentrations of 0.25 and 0.75 g/cm^3^, and at a diameter of 8 mm were used as phantoms.

The micro-CT raw dataset was reconstructed by the NRecon® 1.7.5.1 (Skyscan) with ring artifact correction at 3 and beam hardening correction at 20%. Volumetric visualization was achieved with DataView (Skyscan) software. This resulted in images that were 1536 pixels in width and 1536 pixels in height. Micro-CT can provide a 3D visualization of the inner microstructures, as indicated by changes in the X-ray attenuation value of the specimen^22^.

### Histological observation

The tissue sections for light microscope examination were prepared in three steps: dehydration, clearing, and impregnation. Dehydration was carried out in ascending concentrations of ethanol (EtOH): 70% (EtOH) −> 80% (EtOH) −> 90% (EtOH) −> 95 (EtOH) −> 100% (EtOH). The clearing consisted of the removal of the dehydrant with Neo-clear® (Aruimea, Madrid, Spain) in the following order: 100% (Neo-clear) −> 100% (Neo-clear) −> 100% (Neo-clear) −> 100% (paraffin) −> 100% (paraffin). The paraffin-embedded tissue blocks were cut in serial sections of 5 µm, which were then stained with hematoxylin–eosin (H&E). The slides were then scanned with a 3D scanner (PANNORAMIC 250 Flash III; 3DHISTECH, Budapest, Hungary) and examined using slide-viewing software (CaseViewer version 2.0; 3DHISTECH).

### Ultrastructural analysis by SEM

The specimen was immediately placed in a 2.5% glutaraldehyde solution for fixation. Before electron microscopy examination, the specimens were partially encapsulated in a heavy putty impression material and sectioned through their median region without damaging the layered structure. The specimen was removed from the putty impression material and half of the specimen was used for SEM examination, while the other parts were used for histology and TEM examination.

As the specimen is electrically non-conducting, sputter coating with platinum (Pt) was carried out to increase the signal-to-noise ratio and prevent charging of the specimen, which would otherwise occur because of the accumulation of static electric fields before SEM examination (Apreo S®, Thermo Fisher Scientific, Waltham, MA, USA). The secondary electron (SE) detection mode was used for the ultrastructural surface analysis, while the backscattered electron (BSE) detection mode was used to analyze the different phases and their composition based on the differences in atomic number. We examined the samples from the periphery to the core of the specimen with focus points ranging from 9 to 15 points. The surface of the cross-section was thoroughly examined under 500× magnification and areas with representative features were chosen for micrographic and element analysis. The SEM was operated at 10 kV and 65×, 500×, 1000×, 2500×, 5000×, 10,000×, and 20,000× magnifications. To recognize the asserted structures, we employed visual inspection and compared them with the reference structures, and used EDS analysis for elemental identification.

### Chemical composition analysis by EDS

The element composition of the specimens was accessed by using an EDS instrument (XFlash® 6, Bruker) connected to a microscope detector and the ESPRIT® analysis software (Bruker). Region of interest (ROI) for EDS element analysis was carried out at the center, middle, and external surfaces of each specimen. The representative point of each region was chosen and analyzed under a magnification of 10,000×.

The EDS method involved qualitative and semi-quantitative microanalysis, including element distribution mapping. The representative point of each region was chosen and analyzed under a magnification of 10,000×. The mass concentration (C) was classified by the percentage of weight (wt%) and the atomic weight (at%).

### TEM observation

Specimen preparation involved fixation of samples in 2.5% glutaraldehyde for at least three days. For the TEM examination (JEM-1400 Flash®, Jeol Ltd., Tokyo, Japan), the specimen was stripped into a 1 × 1 × 1 mm block, embedded in epoxy resin, and cut into ultrathin sections (70–80 nm). Sections of 1 μm were stained with toluidine blue (TB) and examined under microscope (BX41 Light Microscope®, Olympus Co., Tokyo, Japan). Biological TEM sample preparations were applied to ultrathin sections. The process began with 2.5% GA primary fixation and dehydration with ethanol, then replaced with propylene oxide, followed by epon infiltration and embedding, sectioning, and mounting on specimen grids. TEM (JEM-1400 Flash®, Jeol Ltd.) was used to observe the components present in the specimen with 3000×, 6000×, 10,000× magnifications.

### Statistical analysis

For the chemical composition analysis by EDS, the means and standard deviations (SDs) of the wt% and at% were calculated. The data normal distribution was tested by Shapiro–Wilk test. The differences were tested by one-way ANOVA. Statistical analyses were carried out using SPSS version 25.0 (IBM Corp., Armonk, NY, USA). *P* < 0.05 was considered statistically significant.

### Ethical approval

The study protocol complied with the principles of the Declaration of Helsinki and was approved by the Seoul National University Institutional Review Board (S-D20220023). All methods were performed in accordance with the relevant guidelines and regulations. All patients were informed of the surgical procedure with the potential risks and benefits, and an informed consent was obtained to receive the treatment and to be included in the study.

## Results

### Patient characteristics and demographic data

Twenty patients with a total of twenty-two specimens were eligible for the study after the inclusion and exclusion criteria. The patient and treatment data are presented in Supplementary Table S1. The average age was 36.52 ± 15.79, ranging from 7 to 68 years old. There were 8 (40%) male patients and 12 (60%) female patients.

## Sialolith specimen data

The anatomical sites in which the specimen was located are presented in Supplementary Table S1. In 19 patients (95%) out of 20 patients, the sialoliths were located in the submandibular salivary gland. Out of 22 sialolith specimens, ten cases presented on the left submandibular salivary gland (45.45%), while eight cases presented on the right side (36.36%) and three cases on both sides of the submandibular salivary glands (13.63%). Only one case presented a sialolith on the left parotid salivary gland (4.24%). Regarding their number of occurrences, single stones were found in 15 cases (75%), two stones in three cases (15%), and two cases had more than two sialoliths (10%).

### Micro-CT analysis

**Figure 1:**
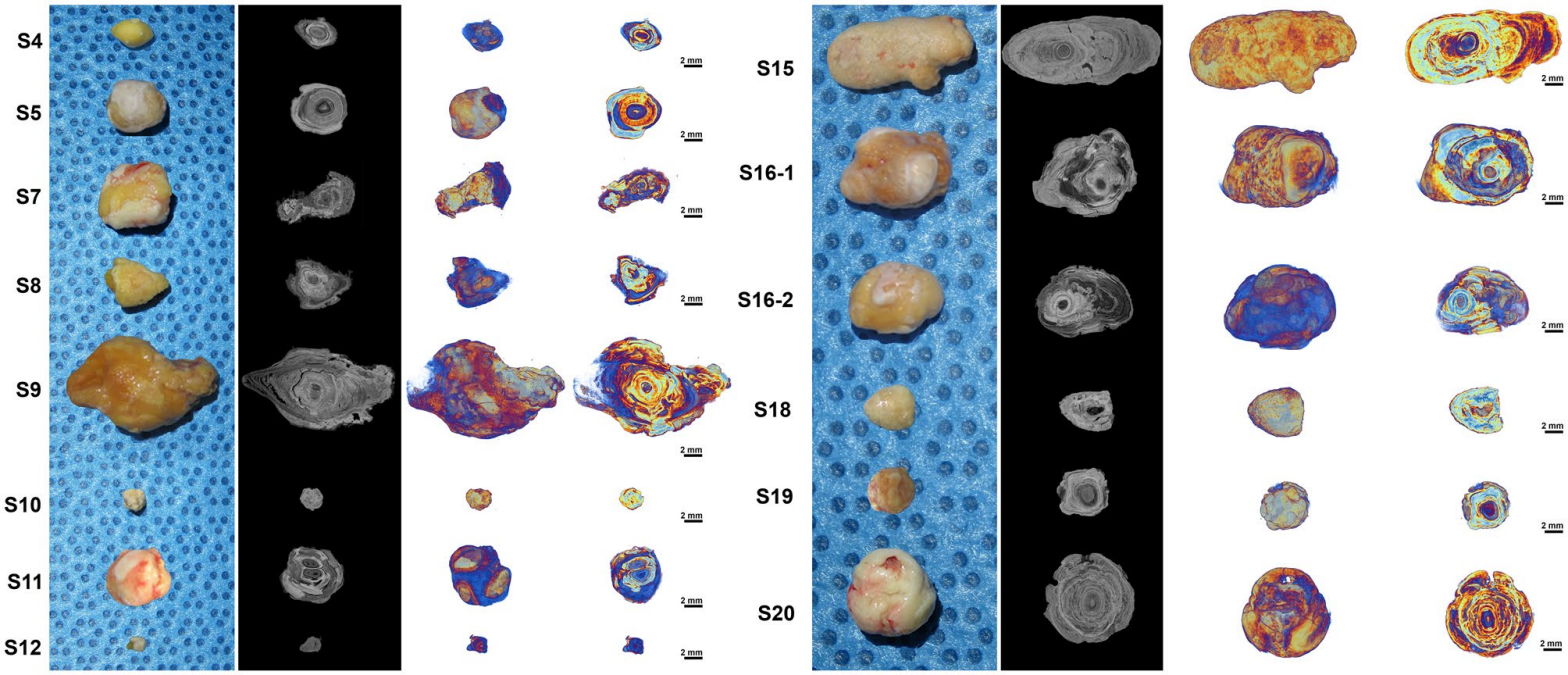
Representative clinical, 2D micro-CT cross-section, and 3D reconstructed images of sialoliths. The micro-CT shows that the sialolith is mainly composed of three layers: the central poorly calcified nidus area surrounded by a highly mineralized zone, and the peripheral multilayered uncalcified zone. Brighter regions represent higher mineralization and dark regions represent organic substances. Scale: 2 mm.

**Figure 2:**
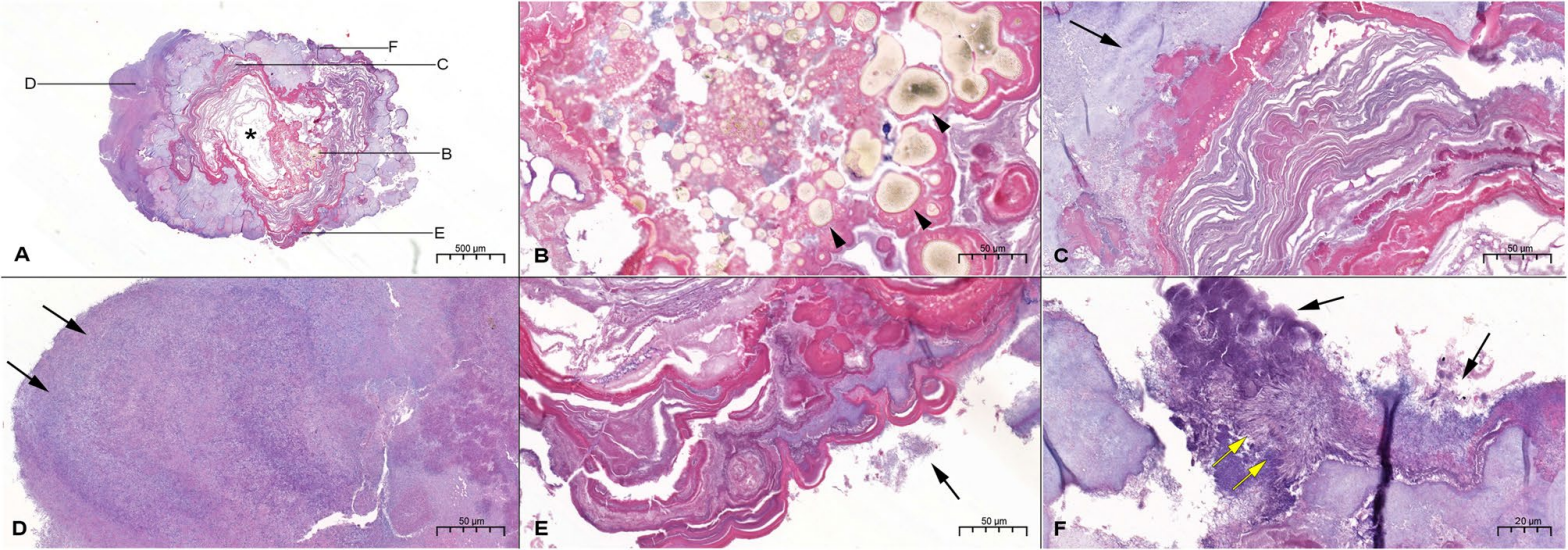
Representative histological findings of sialolith found in the Wharton’s duct orifice (S2) show a poorly mineralized single core, (H&E staining, 4×). Scale bar = 500 μm (**A**). The vesicular structures found at the central nidus area show exosomes (black arrowhead), (H&E staining, 20×). Scale bar = 50 μm (**B**). Highly mineralized nodules were found in the outer layers of the central nidus area (black arrow), (H&E staining, 20×). Scale bar = 50 μm (**C**). The peripheral layers show bacterial colony, which suggests a secondary infection (black arrows), (H&E staining, 20×). Scale bar = 50 μm (**D**). Alternating irregular patterns of highly mineralized nodules were found in the outer layers of the central nidus area (black arrow), (H&E staining, 20×). Scale bar = 50 μm (**E**). Bacterial colony at the peripheral layer (yellow arrows), (H&E staining, 20×). Scale bar = 50 μm (**F**).

The micro-CT scanning allowed 3D visualization of the specimen (Fig. 1). 3D reconstruction video of Fig. 2 is included as supplementary video S2. The sialolith specimens showed an onion skin-like layered structure with alternating radiodense (highly mineralized) and radiolucent (organic) layers in both submandibular and parotid gland sialoliths. The micro-CT images clearly defined a single central nidus in 11 sialoliths (S4, S5, S8, S9, S12, S15, S16-1, S16-2, S18, S19, S20), four central niduses in a sialolith (S7), two central niduses in a sialolith (S11), and an obscure central nidus in a small sialolith (S10). The central nidus in sialolith cores displayed lower degrees of mineralization compared to the surrounding layers as inferred from the brighter and darker contrast in the radiographic data. The sialoliths had nearly spherical (S4, S8, S16-1, S19, S20), oval (S5, S15, S16-2), or asymmetrical (S7, S11, S18) shapes. Apart from the shapes, there were concentric growth patterns (S4, S5, S15, S19) and irregular ones (S7, S8, S9, S11, S16-1, S16-2, S18, S20). However, in many situations, variation between these two types of growth patterns was observed.

### Histological observation

The decalcified microsections showed the organic components of sialolith in the central nidus area and the peripheral multilayer zone in the absence of calcium apatite crystals of intermediate compact zone (Suppl. Figs. S3A–F). And the decalcified microsections of a small sialolith reveal unmineralized central nidus and thick coat of peripheral multilayer zone (Suppl. Figs. S4A–F).

Semi-decalcified microsections of a sialolith obtained from left Wharton’s duct orifice in a 38-year-old female patient (S2) showed a representative feature of sialolith, exhibiting both of poorly mineralized central nidus and partly decalcified matrix of calcium apatite (Figs. 2A–C). Numerous vesicular structures found at the central nidus area show a feature of exosome in variable size, which became enlarged and occasionally fused with each other (black arrowhead) (Fig. 2B). The mineralized matrix was found in the vicinity of the eosinophilic matrix of central nidus (black arrow) (Fig. 2C). The thick bacterial colonies were frequently found on the surface of peripheral multilayer zone (black arrows) probably due to the secondary infection (Figs. 2D–F).

Representative histological findings of a decalcified sialolith obtained from found in the right Wharton's duct orifice in a pediatric patient (S13) had an enlarged central nidus core filled with amorphous eosinophilic materials (white arrows) (Suppl. Figs. S5A–B). Laminated structures of peripheral multilayer zone and circular compact zone were gradually destroyed by the expanding eosinophilic materials (black arrows) of central nidus (Suppl. Figs. S5C–F).

### SEM findings of sialolith

**Figure 3:**
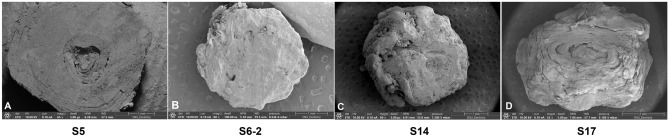
Scanning electron microscopy (SEM) images of four exemplary sialoliths found in the hilar portion of the SMGs at 50× magnification. Sialolith from the left SMG in a 24-year-old female patient (S5) (**A**). Recurrent sialolith in a 50-year-old female patient from the right SMG (S6-2) (**B**). Sialolith in a 48-year-old female patient from the right SMG (S14) (**C**). Recurrent sialolith in a 47-year-old male patient from the left SMG (S17) (**D**).

Surface morphology of SEM observations of the sialolith cross-sectioned are displayed in Fig. 3. The sialolith found in the hilar portion of the left SMG in a 24-years old female patient (S5) with 05 focused points of interest showed a layered structure with a single core nidus. Calcium apatite crystals were seen at point 01. A plate-like structure was observed on the point 02. Bacteria were seen on the core nidus area (yellow arrowhead). Calcium apatite structures were seen on points 04 and 05 (Suppl. Fig. S6).

Sialolith obtained from the hilar portion of the right SMG in 50-years-old female patient (S6-2), shows a three-layered structure (Suppl. Fig. S7). At the periphery, a collection of elongated E. coli-like bacterial biofilm cave (yellow arrowheads) is seen with calcium nanoparticles on point 01. The SEM image shows the layered structure of the sialolith (white lines). At the intermediate layers on points 02, 03, and 04, dense hydroxyapatite aggregates are observed with bacterial empty casts (blue arrowheads) at 20,000× magnification. Point 05 shows a more porous structure than points 02, 03, and 04, but rudely hexagonal, fibrous, irregularly shaped hydroxyapatite crystals are observed^23^. Fibrous and irregularly shaped hydroxyapatite crystals show random orientation from point 05. Point 06 shows the irregularly shaped hydroxyapatite occurring in cluster masses or individual crystals (black arrowheads). Points 07, 09, and 11 show a coarser structure of hydroxyapatite crystal aggregation. Points 08 and 14 depict a densely aggregated layer of microscopic mineral masses compatible with octacalcium phosphate^24^. A filament pattern of the inorganic matter compatible with carbonate apatite arranged in different orientations was observed on point 10 (red arrowheads). Calcite-like crystals are seen on point 13 (blue arrowheads). The irregular structure shows platy, rudely hexagonal and irregularly shaped hydroxyapatite crystals in random orientations (F, blue arrows)^23^.

The heterogeneous layered sialolith found in the hilar portion of right SMG in a 48-years-old female patient (S14) from the submandibular salivary gland shows a single core with a loose layer of granule-shaped crystals with long rod-shaped bacteria at the peripheral layer on point 01. At the middle layer, denser microbial product or biofilm was observed on point 02. Points 03, 04, 07 and 08 at the surface of the core show mineral masses compatible with octacalcium phosphate. At the core of the sialolith a hexahedral calcite crystal having a mineral composition compatible with whitlockite (blue arrowhead) is observed in a long rod-shaped bacterial mold on point 05^24^. At the core of the sialolith, less dense hydroxyapatite aggregates are observed on point 06 (Suppl. Fig. S8).

**Figure 4:**
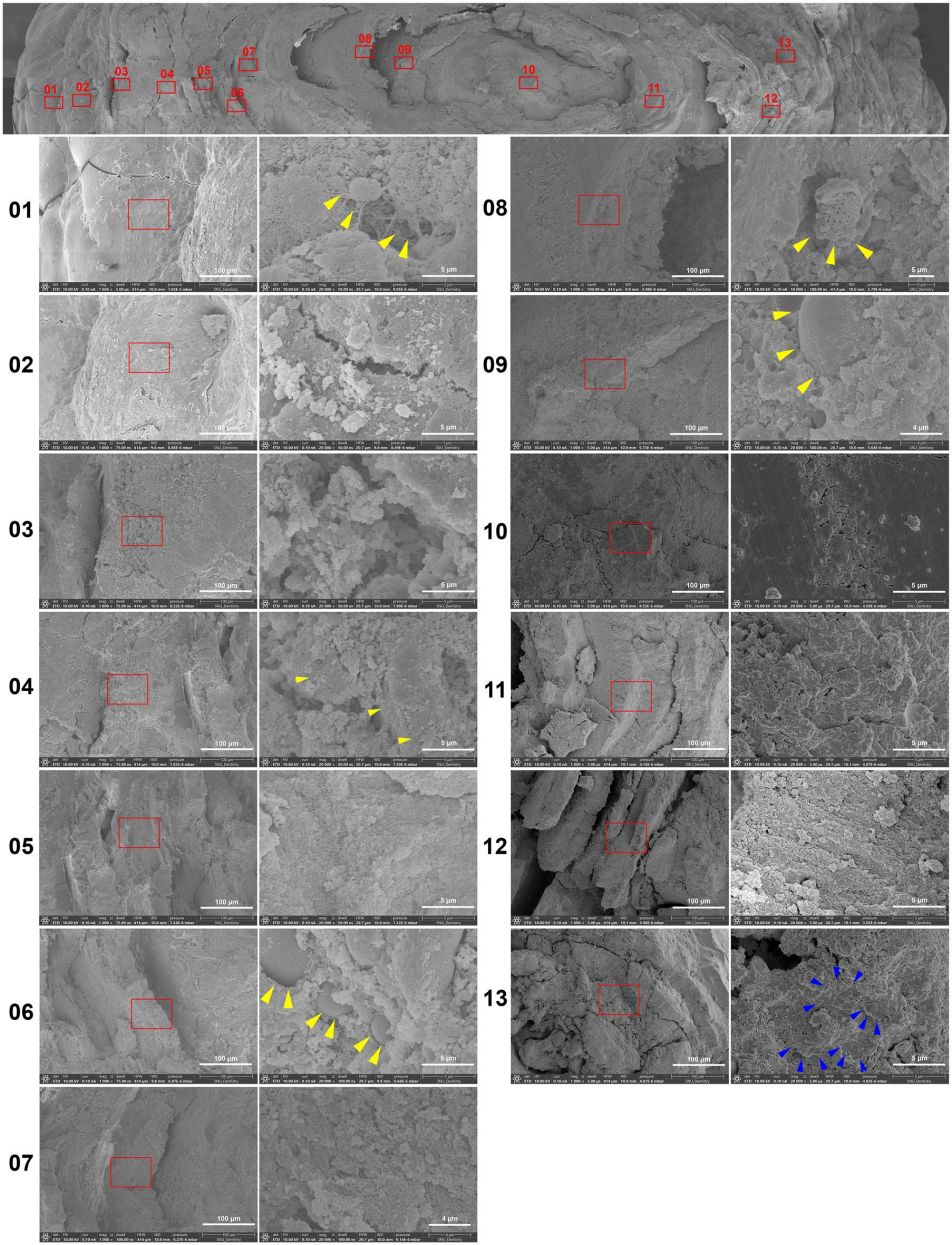
Combined SEM images at 500× magnification of a recurrent sialolith in a 47-year-old male patient from the left SMG (S17) with 13 points of interest. The peripheral layer at point 01 shows cells, cell fibrils, and calcium deposition (yellow arrowhead), magnification 1000×, 20,000×. Calcium crystal deposition on the peripheral layers on points 02 and 03, magnification 1000×, 20,000×. Cells and calcium crystal deposition in the highly mineralized middle layer surrounding the core nidus on points 04–07, magnification 1000×, 20,000×. Caveolae of an atrophied cell shown on point 08, magnification 1000×, 20,000×. Cells on the core nidus area on point 09 (yellow arrowhead), magnification 1000×, 20,000×. The second layer shows calcium apatites with fine granular deposition seen on points 10–12, but cells were not seen, magnification 1000×, 20,000×. The crystallization pattern of calcium hydroxyapatite is shown on point 13 (marked with blue arrowheads), magnification 1000×, 20,000×.

A recurrent sialolith found in the hilar portion of the left SMG in a 47-year-old male patient (S17) from the left submandibular salivary gland was analyzed to observe the cross-section surface at the center with 13 points of interest. The peripheral layer at point 01 shows cells, cell fibrils, and calcium deposition (yellow arrowhead). Calcium crystal deposition on the peripheral layers on points 02 and 03. Cells and calcium crystal deposition in the highly mineralized middle layer surrounding the core nidus on points 04–07. Caveolae of an atrophied cell were seen on point 08. Cells on the core nidus area on point 09 (yellow arrowhead). The second layer showed calcium apatites with fine granular deposition seen on points 10–12, but cells were not seen. The crystallization pattern of calcium hydroxyapatite was depicted on point 13 (marked with blue arrowheads) (Fig. 4).

BSE mode analysis of the recurrent sialolith found in the hilar portion of left SMG in a 47-year-old male patient (S17) showed the concentric pattern of mineralization at 500× and 10,000× magnifications (Suppl. Fig. S9). The fine structure of each layer was threaded with varying sizes and degrees of mineralized globules. The globules had different sizes measuring up to 6 μm. The core of the sialolith consisted of a less mineralized mass at point 04. The surrounding outer layer consisted of a darker and less mineralized structure. The thickness of each layer decreased from the outer layer at the periphery to the core of the sialolith.

### Comparison of elemental analysis at the peripheral, middle, and core regions of sialoliths

The chemical composition of the specimen was analyzed and recorded at the peripheral (P), middle (M), and core (C) layers (Suppl. Figs. S10–13). For the EDS analysis, more traces of chemical elements were found in the peripheral layer of the sialolith compared to the middle and core layers. Ca, C, O, and P were the major elements found in all hydroxyapatite layers of the sialolith. Elements such as Ca, C, O, N, Na, P, Si, and Mg were found in more than two regions. Traces of Cu (wt% 13.39 ± 0.00 and at% 4.62 ± 0.00) and Zn (wt% 10.68 ± 0.00 and at% 3.59 ± 0.00) were found only in the peripheral region of the sialolith (Table 1). Traces of F were found in the periphery and the middle layer of the sialolith but were absent in the core layer. Wt% and at% of Mg were highest in the inner core layer of the specimen (wt% 10.72 ± 7.38 and at% 6.78 ± 4.34) but were not significantly different.

**Table 1:** Elemental composition of the sialolith specimen at the peripheral, middle and core nidus regions.

	Weight percentage (wt%)				Atom percentage (at%)			
	Periphery	Middle	Core	*p* value	Periphery	Middle	Core	*p* value
Ca	19.97 ± 4.36	20.73 ± 4.37	22.15 ± 8.50	0.826	9.58 ± 2.04	9.25 ± 2.53	10.28 ± 4.06	0.817
C	21.19 ± 2.53	40.08 ± 4.79	36.53 ± 4.39	0.585	34.00 ± 5.18	32.09 ± 5.36	30.36 ± 5.59	0.531
O	33.24 ± 13.26	40.08 ± 4.79	36.54 ± 4.39	0.19	39.54 ± 15.93	39.54 ± 15.63	41.61 ± 5.87	0.952
Cu	13.39 ± 0.00	–	–	–	4.62 ± 0.00	–	–	–
F	6.64 ± 0.11	4.39 ± 0.00	–	0.037*	6.81 ± 0.13	4.62 ± 0.00	–	0.048*
N	12.22 ± 8.54	15.71 ± 3.03	15.41 ± 7.41	0.720	15.62 ± 8.60	18.29 ± 3.21	19.06 ± 9.47	0.808
Na	2.22 ± 0.55	2.61 ± 0.00	2.56 ± 0.09	0.531	2.23 ± 0.00	2.23 ± 0.00	2.18 ± 0.09	0.833
P	12.58 ± 6.88	8.10 ± 10.77	12.03 ± 6.31	0.804	11.07 ± 0.00	5.22 ± 6.99	7.42 ± 3.94	0.650
Si	9.12 ± 7.54	10.02 ± 6.66	9.66 ± 6.00	0.978	6.77 ± 6.05	6.49 ± 4.26	5.96 ± 2.99	0.974
Zn	10.68 ± 0.00	–	–	–	3.59 ± 0.00	–	–	–
Zr	7.41 ± 0.00	–	–	–	1.59 ± 0.00	–	–	–
Mg	1.76 ± 0.00	1.76 ± 0.00	10.72 ± 7.38	0.531	1.42 ± 0.00	0.99 ± 0.00	6.78 ± 4.34	0.502
Ca/P	1.59 ± 0.63	2.56 ± 0.41	1.84 ± 1.35	–	0.87 ± 0.00	1.77 ± 0.36	1.39 ± 1.03	–

One-way ANOVA test was used for comparison between groups.

*p < 0.05 were considered statistically significant.

The peripheral layer showed major elements including Ca, C, O, N, Na, P, Si, and Mg. Other elements such as Cu, F, Zn, and Zr were also found. The core of the sialoliths was mostly composed of Ca, C, O, N, Na, P, Si, and Mg.

The EDS mapping showed homologous elemental distribution in sialoliths (Suppl. Figs. S10–13), except in sialolith S5, where a non-homologous elemental distribution of Si and Ca was found in the middle layer (02-M) (Suppl. Fig. S10). The sialolith S17 in supplemental Fig. S14, despite having weight percentages of 20.50, 18.93, 18.76, 18.15, and 29.08%, respectively, the merged distribution map appears predominantly yellow in points 01–05, corresponding to the Ca element. This may be due to several factors, such as the location or distribution of the Ca-containing phase in the sample, or the intensity of X-ray emissions from Ca compared to other elements. Although the mass percentage of Si in points 03 and 04 is 2.33% and 2.89%, respectively, Si may not be visually prominent in the distribution map due to its low concentration in the regions where the SEM–EDS analysis was conducted.

### TEM analysis

**Figure 5:**
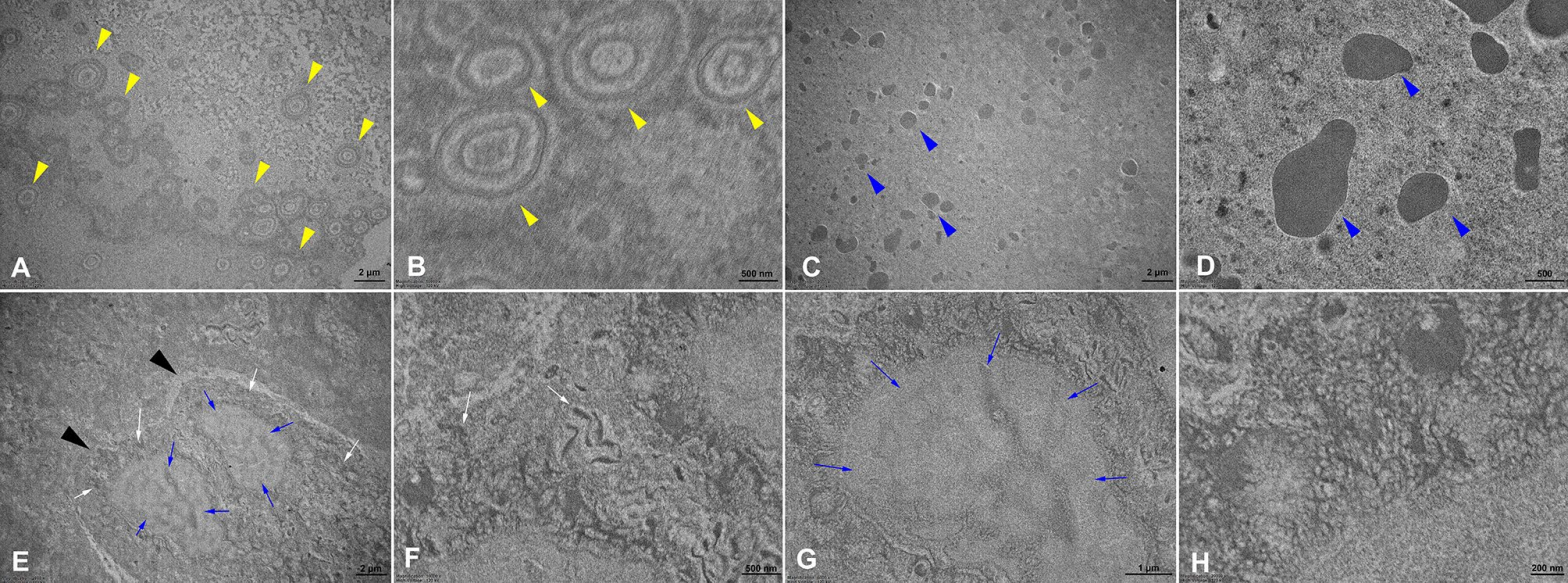
Representative TEM images of sialolith located in the hilum of the SMG in a 48-year-old male patient (S6-1). Bacteria with double membranes are seen on the peripheral layer of the stone (yellow arrowheads), magnification 2000×, 10,000× (**A**–**B**). A homogenous structure of exosome (blue arrowheads), magnification 2000×, 1000× (**C**–**D**). Two epithelial cells (black arrowheads) and a desmosome (white arrow) with the nucleus (blue arrows), magnification 2000×, 10,000× (**E**–**F**). The nucleus and the endoplasmic reticulum of the epithelial cell (blue arrows), magnification 6000× (**G**). The desmosome of the cell in higher magnification, magnification 20,000× (**H**).

For the TEM analysis, highly mineralized/crystalline regions appear dark in the bright field imaging due to staining and/or the diffraction of heavy elements. In sialolith obtained from the hilar portion of the right SMG in 48-years-old female patient (S6-1), many bacteria coated with double layered membrane (white arrow heads) were found in the matrix of central nidus of sialolith (Figs. 5A–B). The membranous vesicles filled with homogenous materials (blue arrow heads) were also found, and then they were identical to the exosome in the extracellular space, where no cells and no cytoplasmic organelles existed (Figs. 5C–D). Two epithelial cells (marked with black arrowheads) containing many intermediate filaments (white arrows) in their cytoplasms and irregular and low density chromatine (blue arrows) in their nuclei were also found at higher magnifications (Figs. 5E–H). The layered appearance of sialolith from the hilar portion of right SMG in 50-years-old female patient (S6-2) showing exosomes (blue arrowheads) in the external lamella with a heterogeneous crystalline needle-like pattern with debris was observed (Suppl. Figs. S14A–D). Deposition of inorganic material and dead cells was also observed (Suppl. Figs. S14E–H).

The TEM images of pediatric sialolith in a 7-year-old female patient (S13), showed the globular structure was dominant (blue arrowheads) in the internal lamellas, while the crystalline pattern was heterogeneous in several outer layers, with some regions of needle-like patterns (yellow arrowheads) (Suppl. Figs. S15A–B). Intracellular components including atrophic mitochondria were also found (white arrows) (Suppl. Figs. S15C–D). Intracellular components, mitochondria and endoplasmic reticulum were also detected (Suppl. Figs. S15E–H).

In the peripheral lamella, a homogenous layer of organic compounds was found in recurrent sialolith from the hilar portion of left SMG in 47-year-old male patient (S17). Hydroxyapatite crystal aggregates were found within the globules and at the outer edges of the globules (Suppl. Figs. S16A–D). Deposition of large single microcrystalline inorganic compounds, such as needle-like filamentary crystals, was arranged in clusters and different directions and clusters of parallelepiped crystals were observed (Suppl. Figs. S16E–H)^25^.

## Discussion

Physiological mineralization is limited to specific sites in the skeletal tissues, including the growth plate cartilages, bone, and teeth. Uncontrolled and pathological mineralization may occur in any soft tissue within the human body^26^. In particular, the calcifications in the articular cartilage, cardiovascular tissues, and kidneys are studied extensively. Although there were numerous publications on sialolithiasis over many years, the exact mechanism involving the genesis of sialoliths is largely unknown.

**Figure 6:**
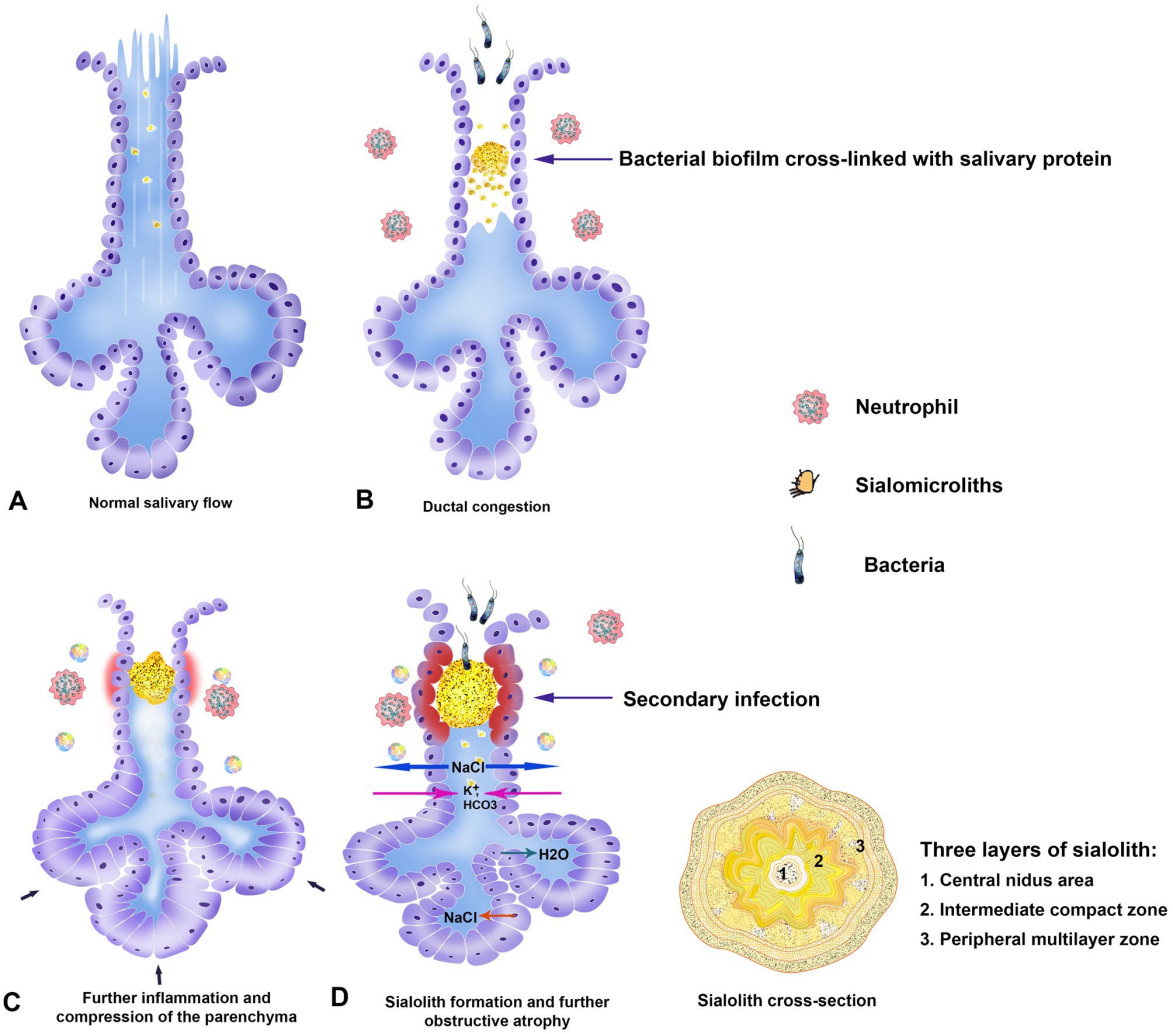
Schematic drawing of the sialolith formation showing the unique three layers. Sialomicroliths are microscopic concretions of the salivary gland and are found in asymptomatic individuals (**A**). During salivary stagnation or secretory inactivity, bacterial infection occurs with biofilm formation cross-linked with salivary protein (**B**). Which causes further inflammation, swelling, sclerosis, and atrophy. At this time neutrophils enter the salivary gland and externalize their NETs, which attract and trap calcium-based crystals such as hydroxyapatite, brushite, and whitlockite and promote the pathogenesis of sialolithiasis (**C**). Secondary infection with oral common bacteria occurs (**D**).

In this study, the histology, micro-CT, SEM and TEM observation revealed unique three layers in the cross section of sialolith, i.e., central nidus zone, intermediate compact zone, and peripheral multilayer zone (Table 2). The central nidus and peripheral multilayer zones were frequently associated with bacterial colonies. According to the distribution of the bacteria found in the central zone, the infection occurred initially in the central nidus, which is able to produce biofilms (Suppl. Fig. S6, point 03). The extensive bacterial colonies found in the peripheral layer seem to be infected secondarily, which produced sialadenitis (Fig. 4) (Suppl. Figs. S7-8). A possible mechanism of sialolith formation is illustrated in Fig. 6 with a schematic illustration of a formed cross-sectioned sialolith (see also in S.I: video S17).

**Table 2:** Summary table of the main morphology, chemical-structures features/results gained from the observed samples.

No	Morphology			Chemical findings			Structure results
	Peripheral	Middle	Central Nidus	Peripheral	Middle	Central Nidus	
S5	Multilayered structure	A plate-like structure	Bacteria	C: 18.55	C: 14.99	C: 19.77	Three layered structures with single core
				O: 33.98	O: 33.8	O: 35.26	
				Na: 2.61	F: 4.39	Na: 3.17	
				Mg: 1.76	Na: 1.8	Mg: 1.76	
				Si: 2.56	Mg: 1.21	P: 14.92	
				P: 17.44	Si: 6.17	Ca: 23.97	
				Ca: 23.1	P: 15.71		
					Ca: 21.93		
S6-2	Long rod-shaped bacterial biofilm with calcium crystal apatites	Highly calcified layer	Calcite-like crystals and hydroxyapatite crystals	C: 21.18	C: 21.47	C: 16.12	Layered structure with single core
				N: 5.77	O: 42.48	N: 10.66	
				O: 24.285	Si: 9.43	O: 38.15	
				Si: 12.615	P: 0.48	Si: 6.22	
				Ca: 19.94	Ca: 26.14	P: 2.57	
				Cu: 13.39		Ca: 26.29	
				Zn: 10.68			
				F: 6.71			
				Zr: 7.41			
S14	Loose layer of granule-shaped crystals and long rod-shaped bacteria	Highly calcified layer	Whitlockite and long rod-shaped bacterial mold	N: 8.99	C: 25.7	C: 27.95	Layered structure with single core
				O: 49.87	N: 21.23	N: 11.62	
				F: 6.56	O: 29.42	O: 38.2	
				Na: 1.83	Si: 18.06	Si: 16.59	
				Si: 8.69	Ca: 16.77	Ca: 5.63	
				P: 7.71			
				Ca: 16.36			
S17	Cells and calcium hydroxyapatite crystallization pattern on the peripheral multilayer	Atrophied cells	Atrophied cells	C: 23.83	C: 23.66	C: 15.89	Layered structure with single core
				N: 21.91	N: 17.73	N: 23.94	
				O: 33.76	O: 38.26	O: 31.09	
				Ca: 20.5	Ca: 18.61	Ca: 29.08	
					Si: 2.33

Morphologically, the central nidus of sialolith is similar to the eye of typhoon, however, contrary to the calm and stormless space of typhoon eye, the central nidus of sialolith is the initiating site for sialolith formation, and contains abundant pathophysiological materials including bacterial biofilm, inflammatory exosomes, and saliva to induce a band-like calcification at the intermediate circular compact zone.

Biofilms are a community of microorganisms that live encased in an extrapolysaccharide (EPS) matrix. Previous studies support the presence of bacteria and/or bacterial products resembling biofilm in sialoliths, which support the hypothesis that biofilm could have a causative effect on lithiasis^27,28^. Biofilms have also been shown to accumulate calcium. Calcium is essential for the cross-linking of alginate in the EPS. Calcium nanoparticles of 500 nm produce calcium apatite from calcium and phosphorus at normal physiological conditions. The interaction of calcium nanoparticles, host cells, and biofilm may serve as a strong scaffold for further calcium deposition and sialolith formation. The main strain of bacterial organisms found in the sialoliths are *Bacillus Subtilis, Streptococcus mitis/oralis, Sterptococcus angionosus, Rothia spp.. Streptococcus constellatus, Streptococcus gordonii, Staphylococcus aureus* and coagulase-negative *Staphylococcus epidermidis*^29,30^. In a previous proteomics study of sialoliths, a group of proteins that defend against different types of external pathogens were identified: lactoperoxidase (LPO), histatin (HTN1), lysozyme (LYZ), and cationic antimicrobial peptide (CAMP)^31^.

Amongst the proteins that are present in the sialolith, the following calcium-binding proteins have a major role in balancing minerals and prevent spontaneous crystallization in the salivary gland and oral cavity: statherin (STATH), histatin (HTN1), calmodulin-like protein 5 (CALML5), basic salivary proline-rich protein 1 (PRB1), proline rich protein HaeIII subfamily 2 (PRH2) and mucin (MUC)^31,32^. STATH has an important role in the saliva which inhibit the spontaneous precipitation of calcium and phosphate salts from the saliva^33^. HTN1 is a histidine-rich peptide that is present in saliva and have anti-inflammation, detoxification, and remineralization effects. The CALML5 protein is involved in calcium-binding, intracellular signaling, and the differentiation of keratinocytes^34^. The PRB1 and PRH2 are acknowledged as modulators of calcium phosphate chemistry, which are selectively absorbed into the hydroxyapatite, and modulate bacterial colonization^35^. Mucins are the main components of mucus, which are high molecular weight glycoproteins. They are categorized into two structurally and functionally different categories such as the secreted type and the membrane-associated type. In a recent study by Schicht et al., MUC8 was found to be secreted into the excretory duct of the salivary glands and released into the saliva of sialolith-bearing glands by immunohistochemistry, dot blot, and ELISA analysis. The study found that MUC8 was deposited in an arranged layer around a single sialolith nuclei and a significantly higher concentration of MUC8 was found in a saliva with sialolith compared to that of saliva without sialolith^32^. MUC8 is known to be upregulated in the context of inflammatory processes, in particular, sialolithiasis is associated with recurrent inflammation. These cross-linking enzymes present in sialolith are thought to have a role in the pathogenic mechanism of hydroxyapatite precipitation in the sialolith (Fig. 4, Suppl. Figs. S6-8).

Regarding factors such as trauma, salivary duct obstruction, habit, systemic metabolism, etc., ductal trauma is thought to be one of the factors that contribute to sialolith formation. However, based on our experience and the literature review, endoscopic sialolith removal does not result in sialolith formation. Regarding the habit, a previous study found a positive association between sialolithiasis and tobacco smoking in a population of ≤ 40 years-old^36^. And considering the systemic metabolism, sialoliths tend to be unilateral, which argues against a systemic metabolism and suggests local factors play major roles^37^.

A comparative study between sialolith, calculus, and a tonsillolith showed that sialolith had the most complex layered structure, with a central core while calculus and tonsillolith did not have a distinct architecture^38^, such as seen on our study (Fig. 1). A further comparative study between different types of calcifications found in the head and neck region could help in understanding the mineralization process of sialolith.

Compared to the previous reports of sialolith structure, the present study added a intermediate circular compact zone between the central nidus area and peripheral multilayered zone. The circular compact zone was conspicuously distinguishable in the observation of micro-CT and SEM (Figs. 1, 3, 4) (Suppl. Figs. S6-9).

The sialolith is a growing stone in salivary duct, which can produce clinical symptoms by obstructing salivary flow and eliciting inflammatory reaction. All sialolith (n = 22) examined in this study showed a single or multiple foci of central nidus(es) as shown in the micro-CT images. And it was found every central nidus contained bacterial or bacteria-associated remnants in the ultrastructural observation of SEM and TEM.

Many authors found vesicular structures in the central core area of sialolith^39,40^, and the present study also found a lot of vesicle structures in the extracellular matrix. By observing TEM images, the ultrastructure of vesicles was identical to the exosomes dispersed in the extracellular space (Figs. 5C–D) (Suppl. Figs. S14C–D). Along with the exosomes, many bacterial structures were found in the central nidus area and intermediate compact zone in SEM and TEM observation. Particularly, TEM showed the compact bi-layered cell membrane of bacteria and prokaryotic nuclear chromatin structure in their cytoplasms (Figs. 5A–B). Therefore, it was supposed that the exosomes abundant in the central nidus area were derived from inflammatory cells, monocytes, neutrophils, etc., recruited for innate immunity against the primary bacterial infection.

The presence of circular compact zone between the central nidus and peripheral multilayer zones is another hallmark of sialolith. The exosomes in the organoid matrix of central nidus may contain different cytokines and enzymes, which are able to elicit inflammatory stimulation and to induce the calcification/ossification processes at the outside of central nidus area. Therefore, it is suggested that the band-like calcification of intermediate circular compact zone is a characteristic enough to explain the mechanism of sialolith formation.

Many epithelial cells were found in the peripheral multilayer zone in TEM (Fig. 4). The epithelial cells were almost atrophic with many irregular vesicles and endoplasmic reticulum structure in their cytoplasms, and their nuclear chromatins were relatively homogenous with low density. Therefore, it was thought that the exfoliated salivary epithelial cells could facilitate the growth of sialolith by repeated deposition of salivary materials and subsequent calcification in the peripheral multilayer zone.

The present study demonstrated that the central nidus area was filled with organoid materials which was poorly calcified, and contained many bacteria in loosely arranged calcium apatite matrix. The bacteria, which were almost atrophic and surrounded by calcified calcium apatite crystals, were consistently found in the intermediate compact zone (Suppl. Figs. S7-8). However, it was hypothesized that the central nidus area of sialolith contains different chemical materials derived from inflammatory exosomes, saliva, and bacterial biofilm to induce the mineralization of calcium apatite crystals necessary for the deposition of the intermediate compact zone. As the bigger sialoliths had the increased size and number of central nidus than the smaller ones in this study, it was also suggested that not only the peripheral multilayer zone but also the central nidus and intermediate circular compact zones are gradually growing in their volume.

The main elements of sialoliths are Ca, P, and O. A small amount of Cu, F, Zn, and Zr were also detected. A study using Fourier transform infrared (FTIR), FT-Raman, and fluorescence spectroscopic techniques showed the ratio of the major elements Ca and P to be 7:3^41^. In another study using X-ray microanalysis, the component elements of sialoliths were Ca, O, S, and Na. The Ca and P ratio was calculated to be 1.60–1.89^42,43^. The chemical constitution of each specimen varied from one to another. The main inorganic components of sialoliths are reported to be apatite (Ca_10_(PO_4_)_6_(OH)_2_), whitlockite (Ca_3_(PO_4_)_2_), and brushite (CaHPO_4_ 2H_2_O). Other components include weddellite and octacalcium phase.

In the EDS analysis on the surface of sialolith cross-section, the Ca/P ratio was the highest in the intermediate compact zone (1.77), and followed by the central nidus area (1.39) and the peripheral multilayer zone (0.87). Therefore, it is suggested that the calcium apatite of intermediate compact zone has similar chemical structure of calcium hydroxyapatite (Ca_10_(PO_4_)_6_(OH)_2_) rather than whitlockite (Ca_3_(PO_4_)_2_), and brushite (CaHPO_4_ 2H_2_O). However, further investigation is required to identify the chemical structures and properties of sialolith calcium apatite variants.

The present study has been carried out depending on the morphological analysis using micro-CT, histology, SEM, and TEM. Therefore, in order to elucidate the pathogenetic mechanism of sialolith, the molecular biological and chemical investigation should be performed in the following study.

## Conclusion

This study investigated and confirmed the ultrastructural and elemental findings of sialoliths with highly mineralized and less mineralized cores using micro-CT, SEM, EDS, and TEM analysis. Many bacteria and inflammation-related exosomes were found in the central nidus of 22 sialolith observed, and salivary epithelium cells were also detected in the peripheral multilayer zone of sialolith through TEM observation. These data may partly explain the pathogenic mechanism of sialolith formation, indicating bacteria-associated salivary protein coagulation and salivary exfoliated cells-associated increase of sialolith size. Additionally, it was suggested that the intermediate compact zone showing higher Ca/P ratio (1.77) than the other zones is mineralized with calcium apatite similar to Ca_10_(PO_4_)_6_(OH)_2_. Integrating the above scientific results, the formed sialoliths have shown activity in the biological zone with presence of bacteria, inflammatory exosomes, exfoliated salivary epithelial cells besides saliva materials. Furthermore, these components co-operatively underwent the pathogenetic progresses including central nidus formation, induction of intermediate compact zone calcification, and repeated deposition of peripheral multilayer zone subsequently.

### Supplementary Information

Supplementary Information 1.

Supplementary Information 2.

Supplementary Information 3.

Supplementary Information 4.

Supplementary Information 5.

Supplementary Information 6.

Supplementary Information 7.

Supplementary Information 8.

Supplementary Information 9.

Supplementary Information 10.

Supplementary Information 11.

Supplementary Information 12.

Supplementary Information 13.

Supplementary Information 14.

Supplementary Information 15.

Supplementary Information 16.

Supplementary Information 17.

Supplementary Information 18.

## Data Availability

The datasets generated during and/or analyzed by the authors during this study are available from the corresponding author on reasonable request.
