# Author Correction: Identification of biological components for sialolith formation organized in circular multi-layers

**DOI:** 10.1038/s41598-023-41386-w

**Published:** 2023-08-30

**Authors:** Buyanbileg Sodnom-Ish, Mi Young Eo, Yun Ju Cho, Mi Hyun Seo, Hyeong-Cheol Yang, Min-Keun Kim, Hoon Myoung, Suk Keun Lee, Soung Min Kim

**Affiliations:** 1 Department of Oral and Maxillofacial Surgery, School of Dentistry, Dental Research Institute, Seoul National University, Seoul, Korea; 2 Department of Dental Biomaterials Science, School of Dentistry, Dental Research Institute, Seoul National University, Seoul, Korea; 3 Department of Oral and Maxillofacial Surgery, College of Dentistry, Gangneung-Wonju National University, Gangneung, Korea; 4 Department of Oral Pathology, College of Dentistry, Gangneung-Wonju National University, Gangneung, Korea

Correction to: *Scientific Reports* 10.1038/s41598-023-37462-w, published online 28 July 2023

In the original version of this Article Min-Keun Kim was incorrectly affiliated with ‘Department of Oral and Maxillofacial Surgery, School of Dentistry, Dental Research Institute, Seoul National University, Seoul, Korea’.

The correct affiliation is listed below:

Department of Oral and Maxillofacial Surgery, College of Dentistry, Gangneung-Wonju National University, Gangneung, Korea.

And Hoon Myoung was incorrectly affiliated with ‘Department of Oral and Maxillofacial Surgery, College of Dentistry, Gangneung-Wonju National University’.

The correct affiliation is listed below:

Department of Oral and Maxillofacial Surgery, School of Dentistry, Dental Research Institute, Seoul National University, Seoul, Korea.

The original Article has been corrected.

