# Supplementary material for: Identification of biological components for sialolith formation organized in circular multi-layers
Source: Sci Rep. 2023 Jul 28;13:12277. doi: 10.1038/s41598-023-37462-w (PMC10382579; doi:10.1038/s41598-023-37462-w)
Supplement: Supplementary file 1 — Supplementary Information 1. [file 41598_2023_37462_MOESM1_ESM.docx]

**Supplementary table S1.** Patient demographic information.

| No | Sex | Age | PMH | Chief complaint | Location | Anesthesia | Treatment |
| --- | --- | --- | --- | --- | --- | --- | --- |
| S1 | F | 22 | ~ | Impacted third molar | Hilar portion of right SMG | GA | Sialoendoscopy and surgical extraction of #18,38,48 teeth |
| S2 | F | 38 | Hypothyroidism | ~ | Left Wharton’s duct orifice | GA | Sialolithotomy |
| S3 | F | 34 | ~ | Impacted third molar | Hilar portion of left SMG | GA | Sialolithotomy and surgical extraction of #18,48 teeth |
| S4 | F | 21 | ~ | Pain and swelling | Hilar portion of right SMG | GA | Sialolithotomy and surgical extraction of #38,48 |
| S5 | F | 24 | ~ | Discomfort | Hilar portion of left SMG | GA | Sialolithotomy and ductoplasty |
| S6-1 | F | 48 | ~ | Pain and swelling | Hilar portion of right SMG | ~ | Spontaneously came out |
| S6-2 | F | 50 | ~ | ~ | Hilar portion of right SMG | ~ | Sialoendoscopy and ductoplasty on right SMG |
| S7 | F | 20 | ~ | Pain and swelling | Hilar portion of SMG (both sides);  tonsils (both sides) | GA | Sialolithotomy, tonsillectomy |
| S8 | M | 55 | ~ | Pain and swelling | Left SMG (left Warton’s duct) | IV | Sialolith removal |
| S9 | M | 63 | HCV, HTN | Pain and swelling | Left Wharton's duct orifice | IV | Sialolithotomy |
| S10 | F | 28 | ~ | Recurrent tender swelling | Left Wharton's duct orifice | IV | Sialolithotomy (left SMG) |
| S11 | F | 28 | ~ | Impacted third molar | Hilar portion of right SMG | IV | Sialolithotomy (right SMG) |
| S12 | F | 29 | ~ | Pain and swelling | Left Wharton's duct orifice | ~ | Sialolithotomy (left SMG) |
| S13 | F | 7 | ~ | Pain and swelling | Right Wharton's duct orifice |  | Minor sialolithotomy |
| S14 | F | 48 | ~ | Pain, limited mouth opening | Hilar portion of right SMG | IV | Sialolithotomy (right SMG) |
| S15 | M | 29 | ~ | Swelling | Hilar portion of right SMG | IV |  |
| S16-1; S16-2 | M | 39 | ~ | Pain | Both SMG hilum area | GA | Sialolithotomy, ductoplasty |
| S17 | M | 47 | ~ | Pain and swelling | Hilar portion of left SMG | IV | Sialolithotomy |
| S18 | M | 68 | Hyperlipidemia, allergic rhinitis | Pain and swelling | Left Stensen’s duct orifice | IV | Endoscopic assisted sialolithotomy, ductoplasty |
| S19 | M | 21 | ~ | Pain and swelling | Hilar portion of left SMG | IV | Sialolithotomy |
| S20 | F | 30 | ~ | Discomfort | Hilar portion of right SMG | GA | Sialolithotomy and ductoplasty |

PMH, past medical history; GA, general anesthesia; IV, intravenous sedation; SMG, submandibular salivary gland; HCV, hepatitis C virus; HTN, hypertension
