# Supplementary material for: Identification of biological components for sialolith formation organized in circular multi-layers
Source: Sci Rep. 2023 Jul 28;13:12277. doi: 10.1038/s41598-023-37462-w (PMC10382579; doi:10.1038/s41598-023-37462-w)
Supplement: Supplementary file 2 — Supplementary Information 2. [file 41598_2023_37462_MOESM2_ESM.pptx]

## Slide 1
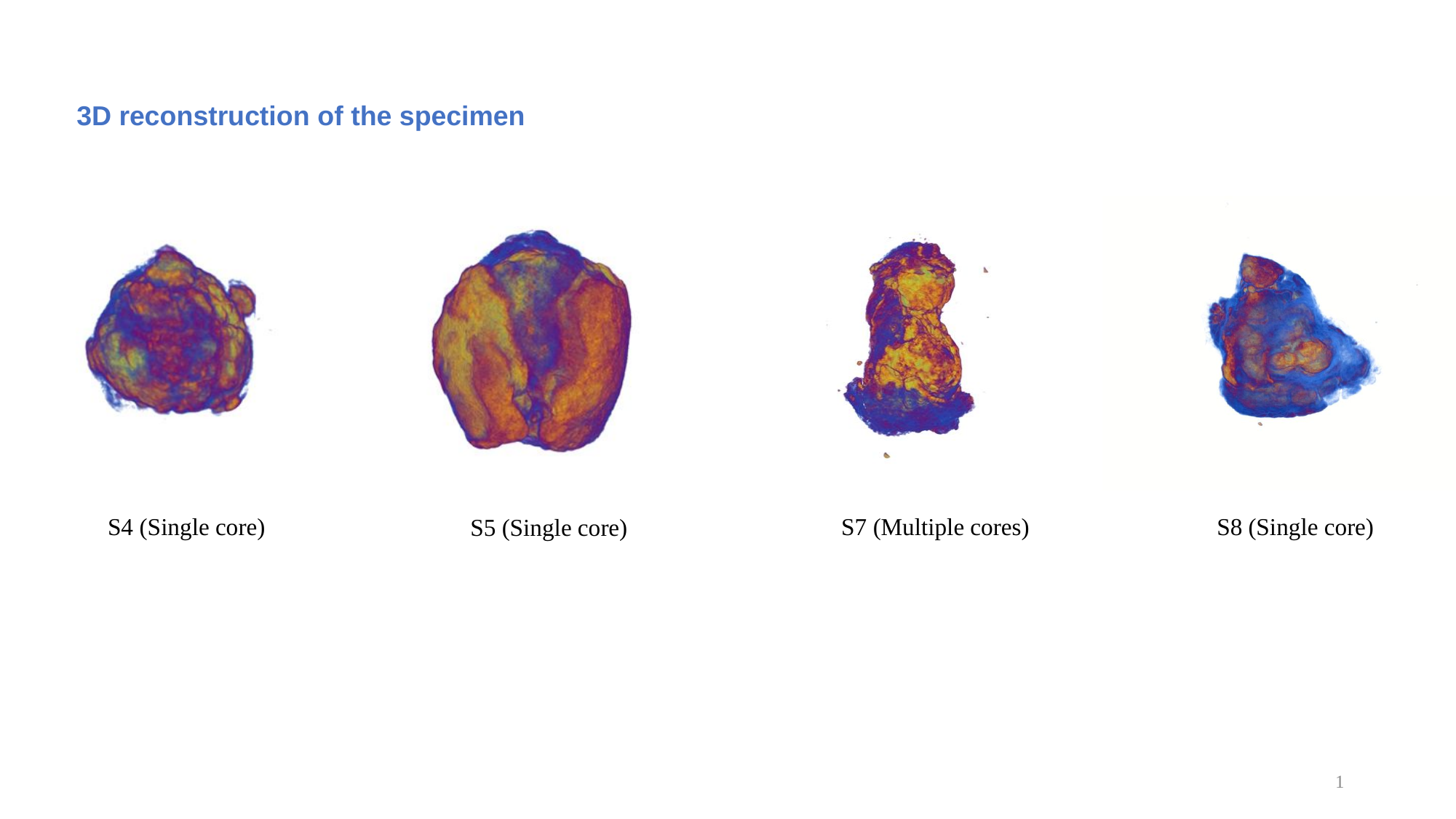

3D reconstruction of the specimen
S4 (Single core)
S7 (Multiple cores)
S8 (Single core)
S5 (Single core)
1

## Slide 2
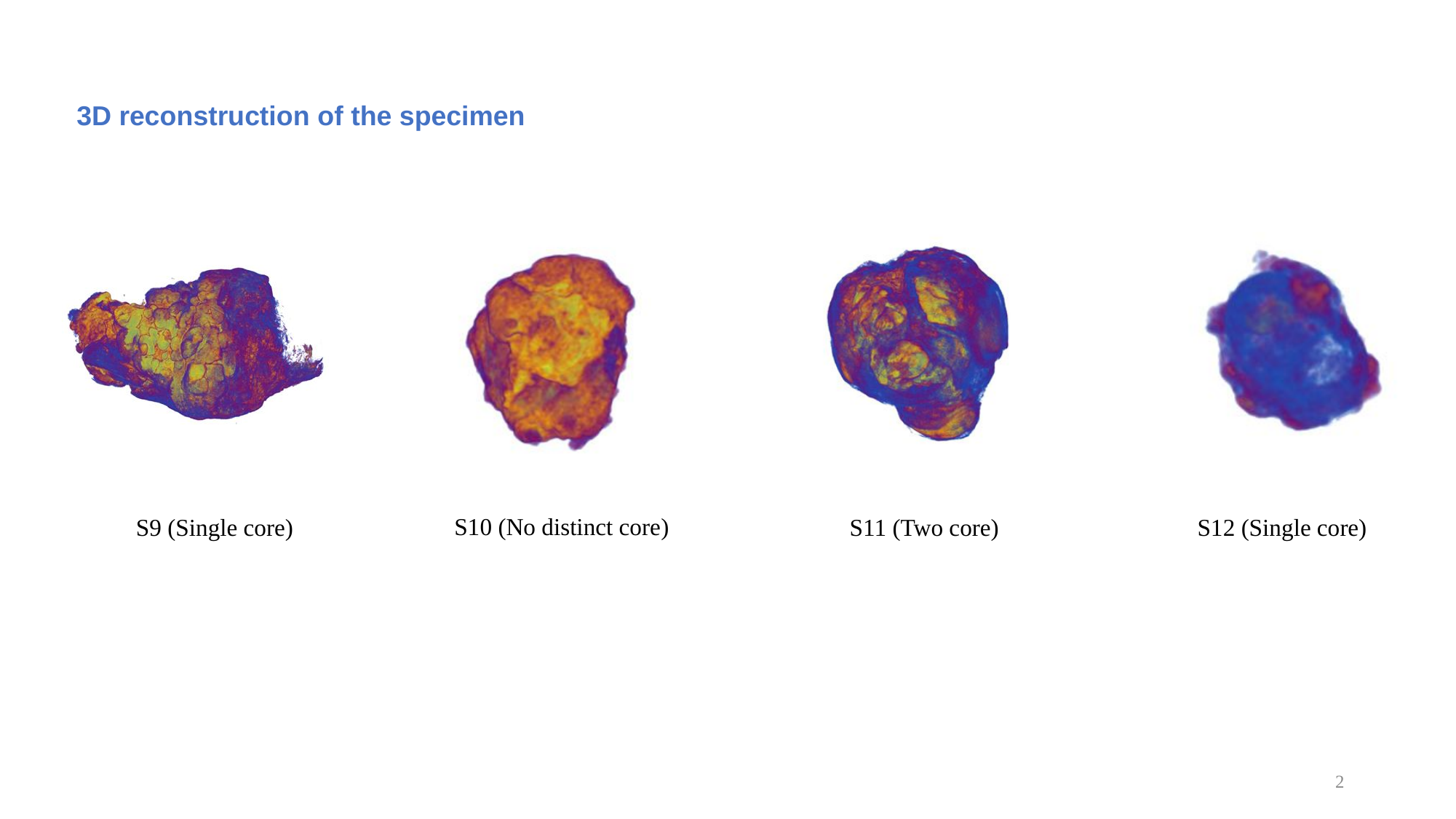

3D reconstruction of the specimen
S10 (No distinct core)
S9 (Single core)
S11 (Two core)
S12 (Single core)
2

## Slide 3
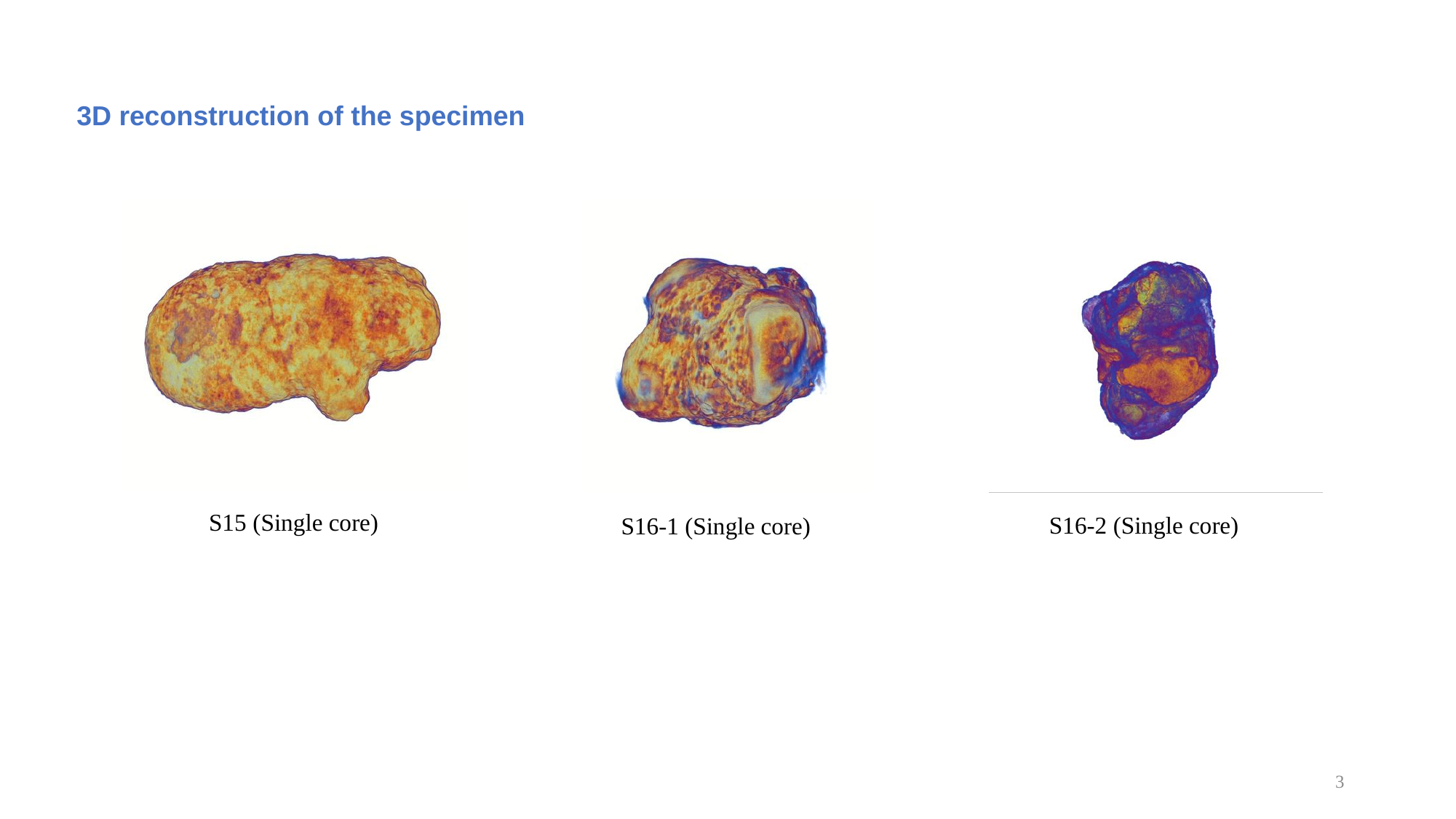

3D reconstruction of the specimen
S15 (Single core)
S16-2 (Single core)
S16-1 (Single core)
3

## Slide 4
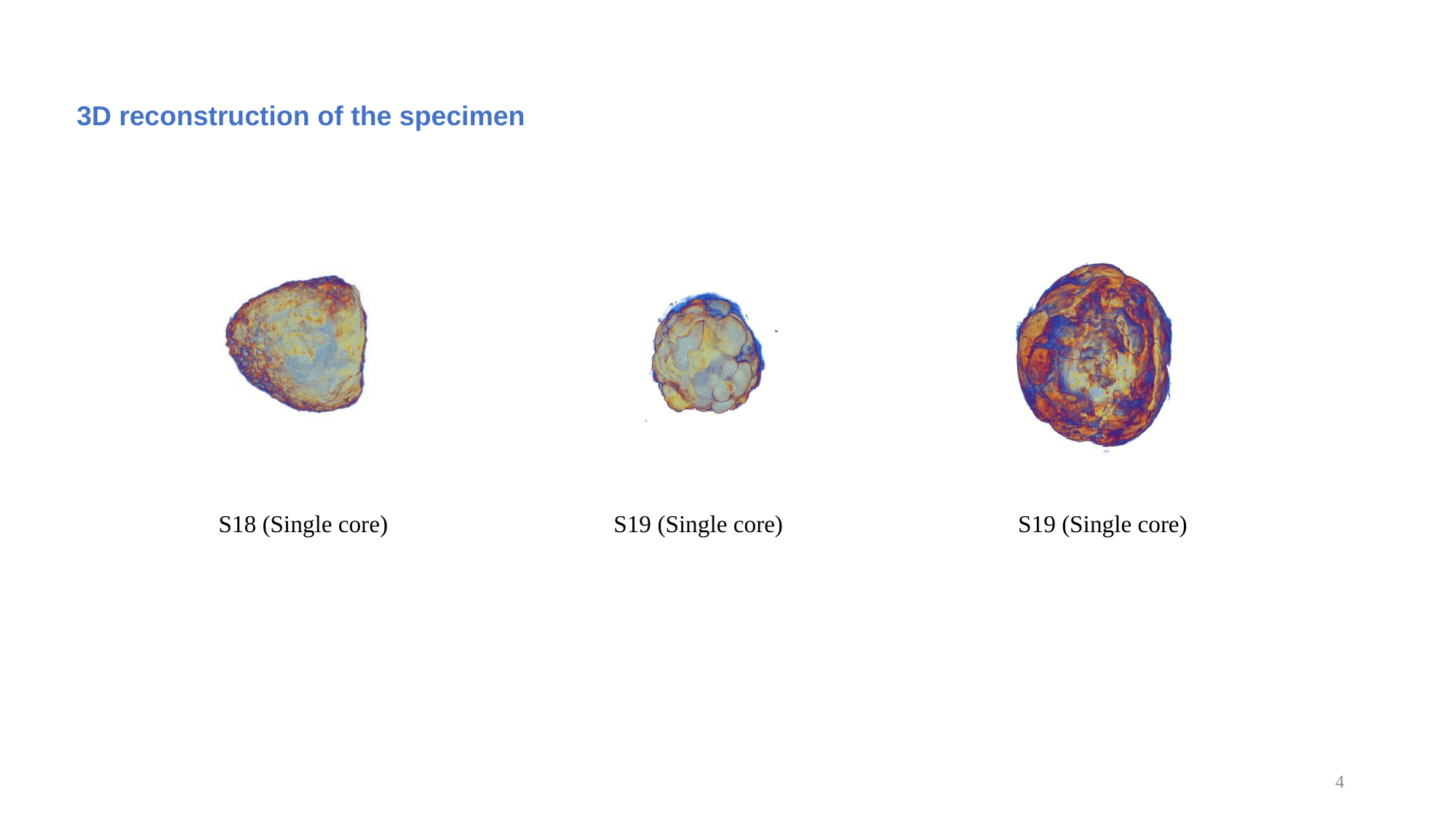

3D reconstruction of the specimen
S18 (Single core)
S19 (Single core)
S19 (Single core)
4
