# Supplementary figures and images for: Identification of biological components for sialolith formation organized in circular multi-layers
Source: Sci Rep. 2023 Jul 28;13:12277. doi: 10.1038/s41598-023-37462-w (PMC10382579; doi:10.1038/s41598-023-37462-w)

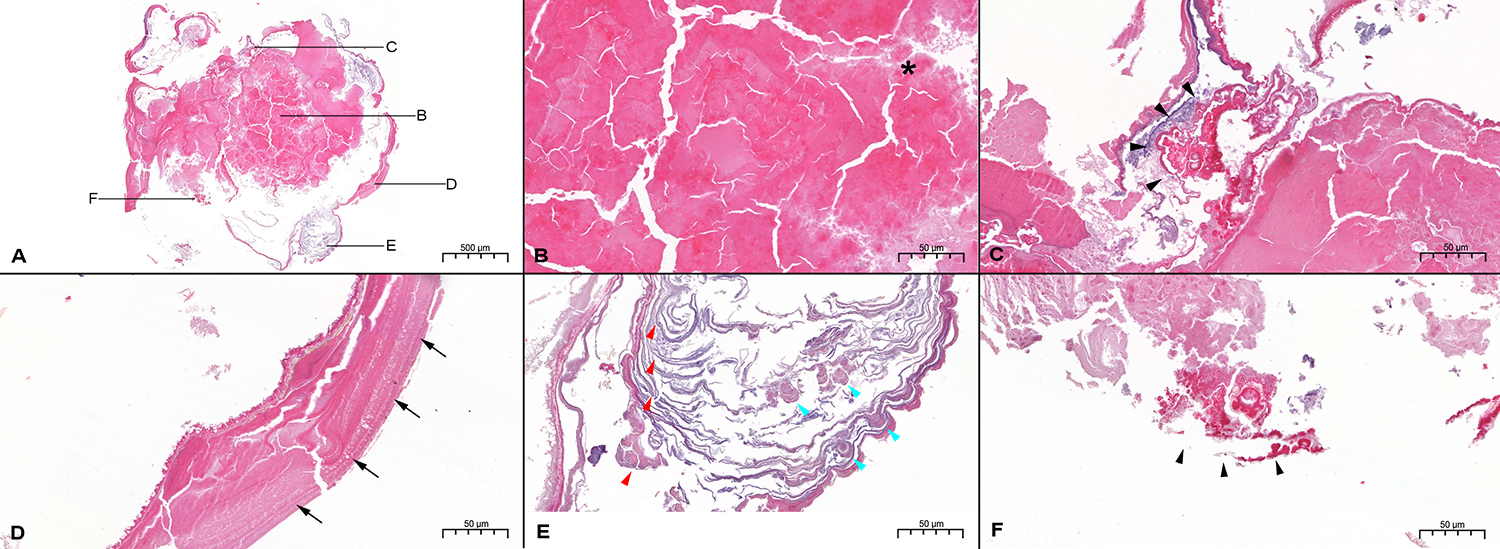

Supplement: Supplementary file 3 — Supplementary Information 3. [file 41598_2023_37462_MOESM3_ESM.jpg]

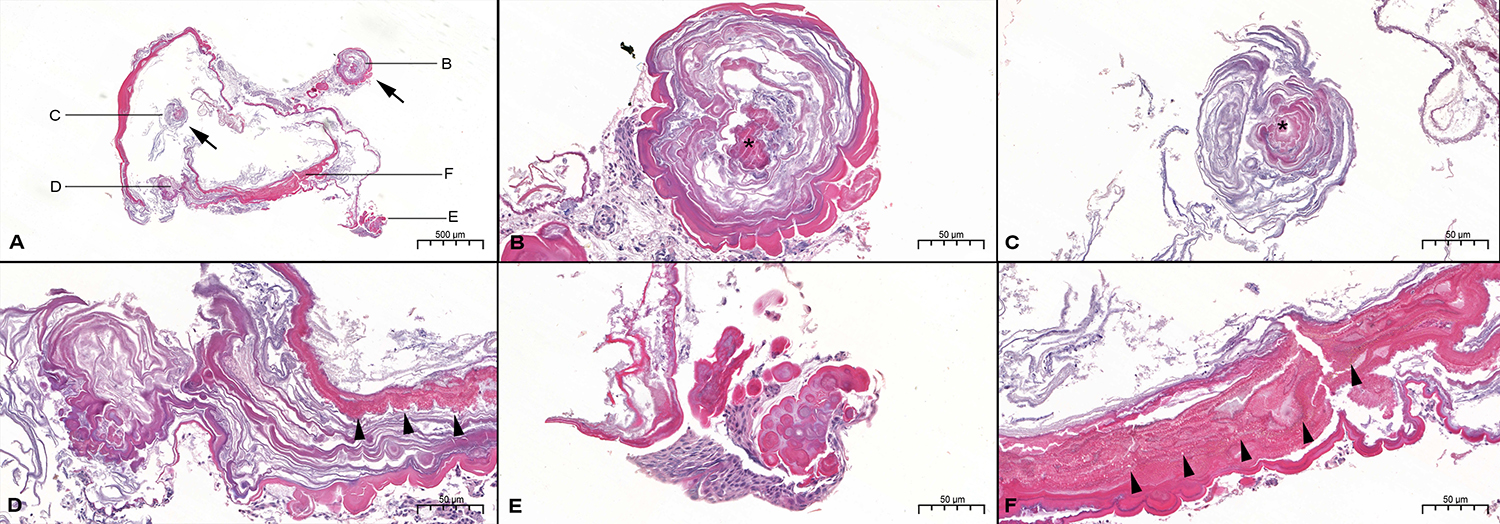

Supplement: Supplementary file 4 — Supplementary Information 4. [file 41598_2023_37462_MOESM4_ESM.jpg]

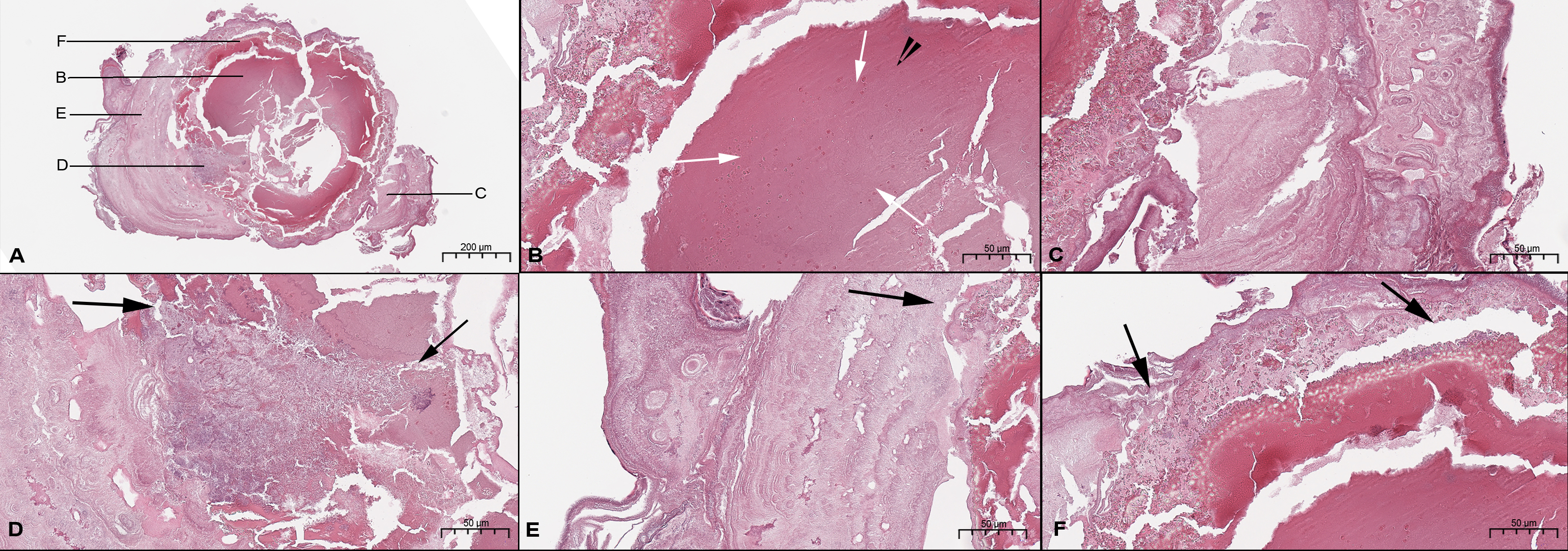

Supplement: Supplementary file 5 — Supplementary Information 5. [file 41598_2023_37462_MOESM5_ESM.jpg]

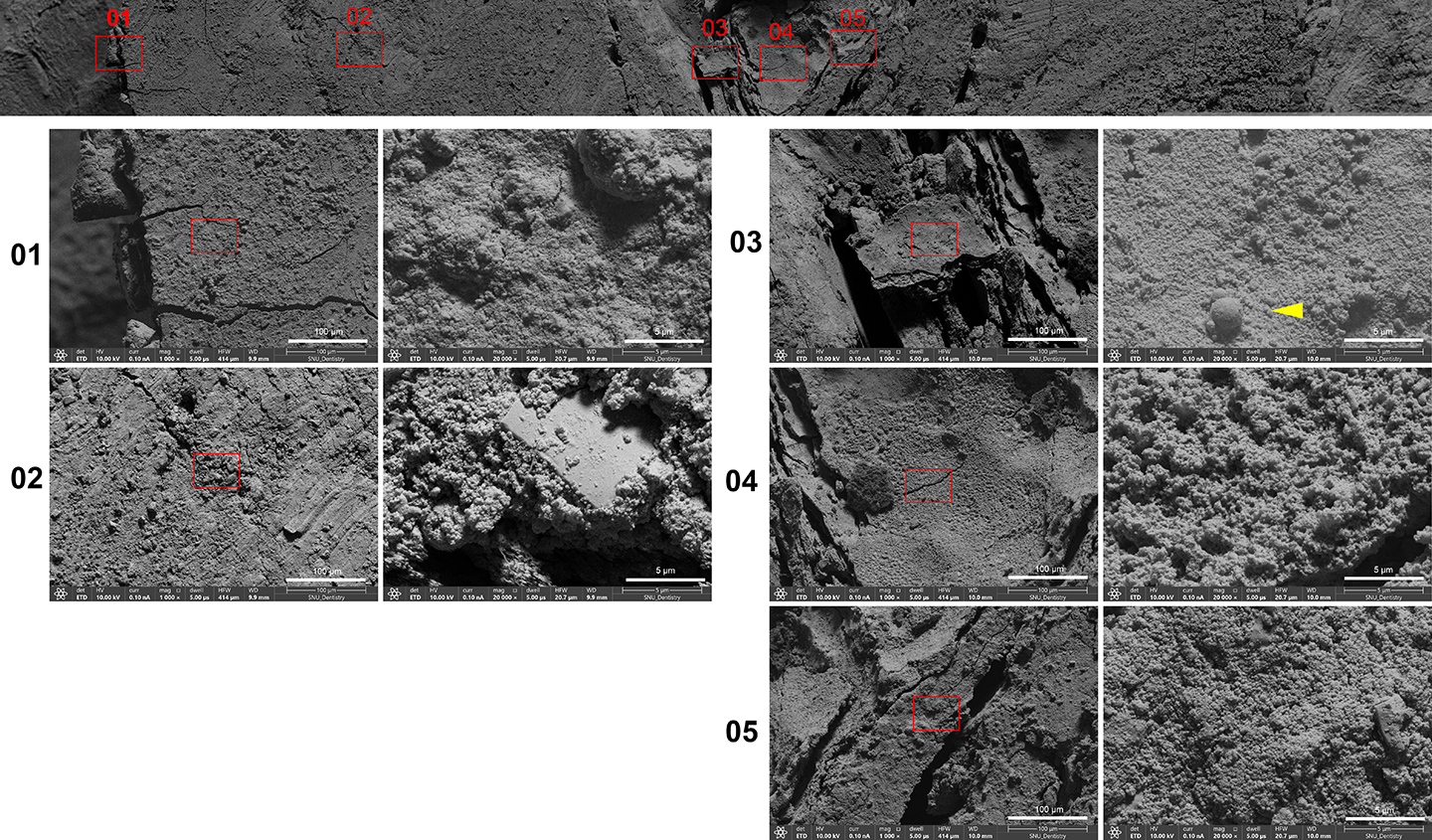

Supplement: Supplementary file 6 — Supplementary Information 6. [file 41598_2023_37462_MOESM6_ESM.jpg]

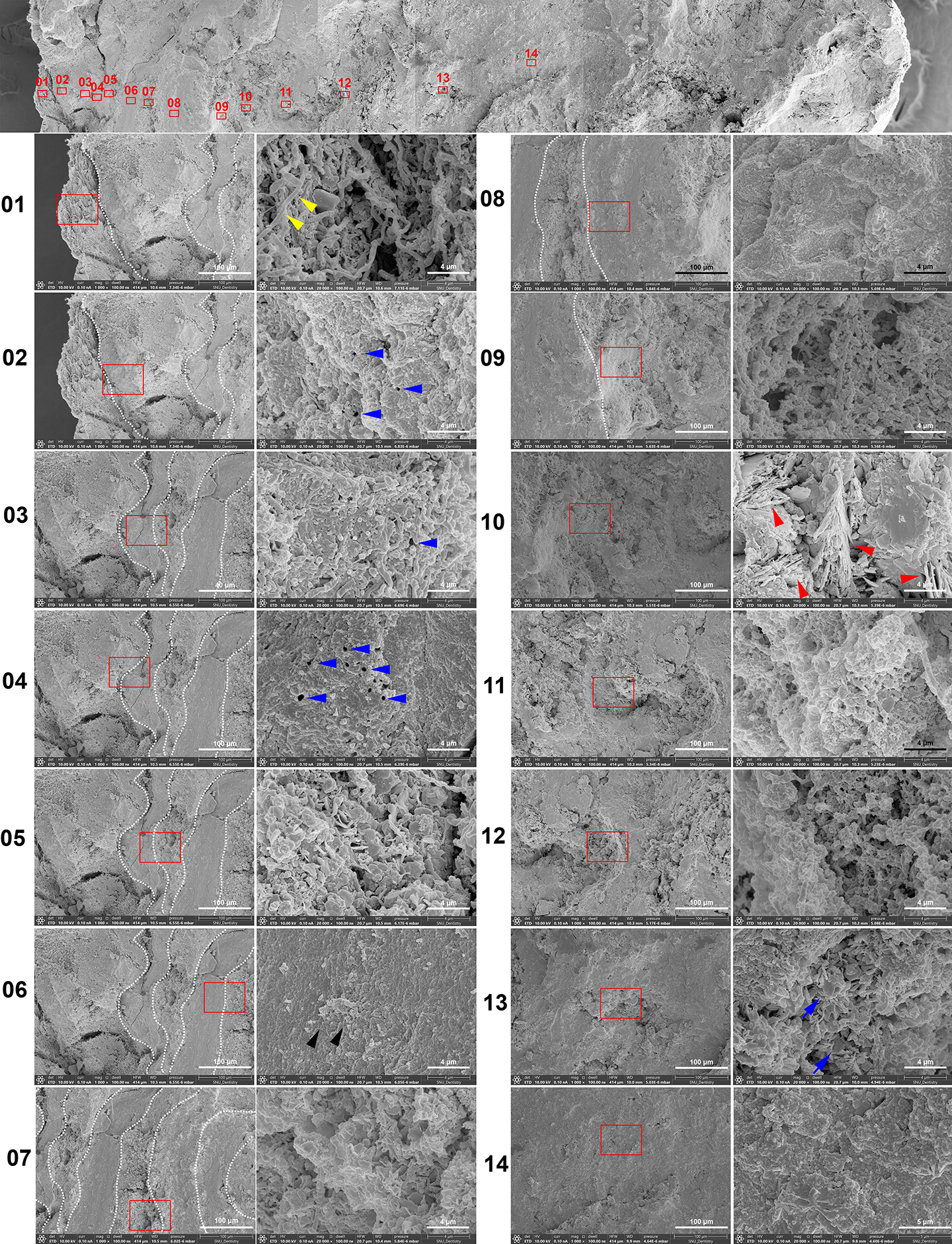

Supplement: Supplementary file 7 — Supplementary Information 7. [file 41598_2023_37462_MOESM7_ESM.jpg]

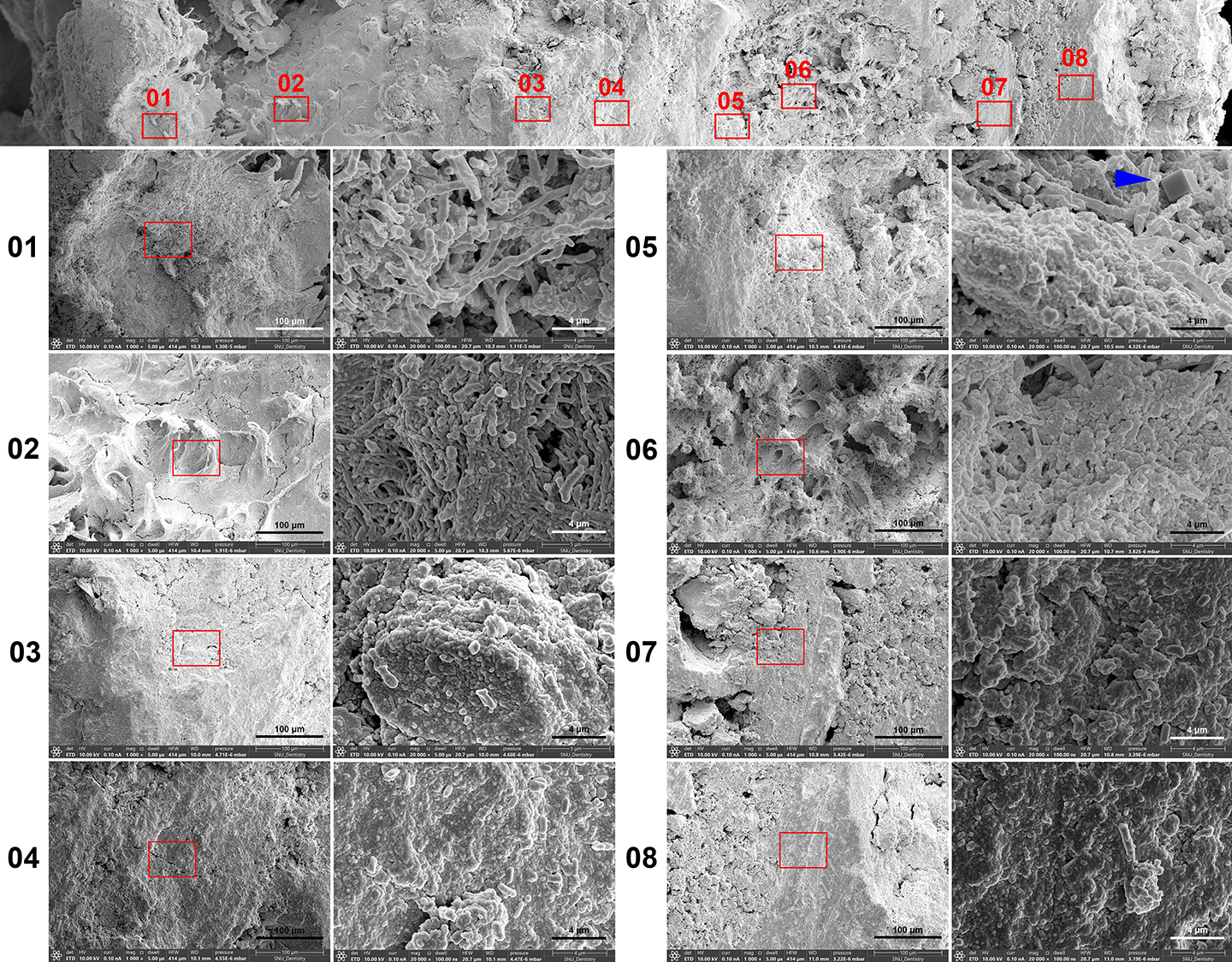

Supplement: Supplementary file 8 — Supplementary Information 8. [file 41598_2023_37462_MOESM8_ESM.jpg]

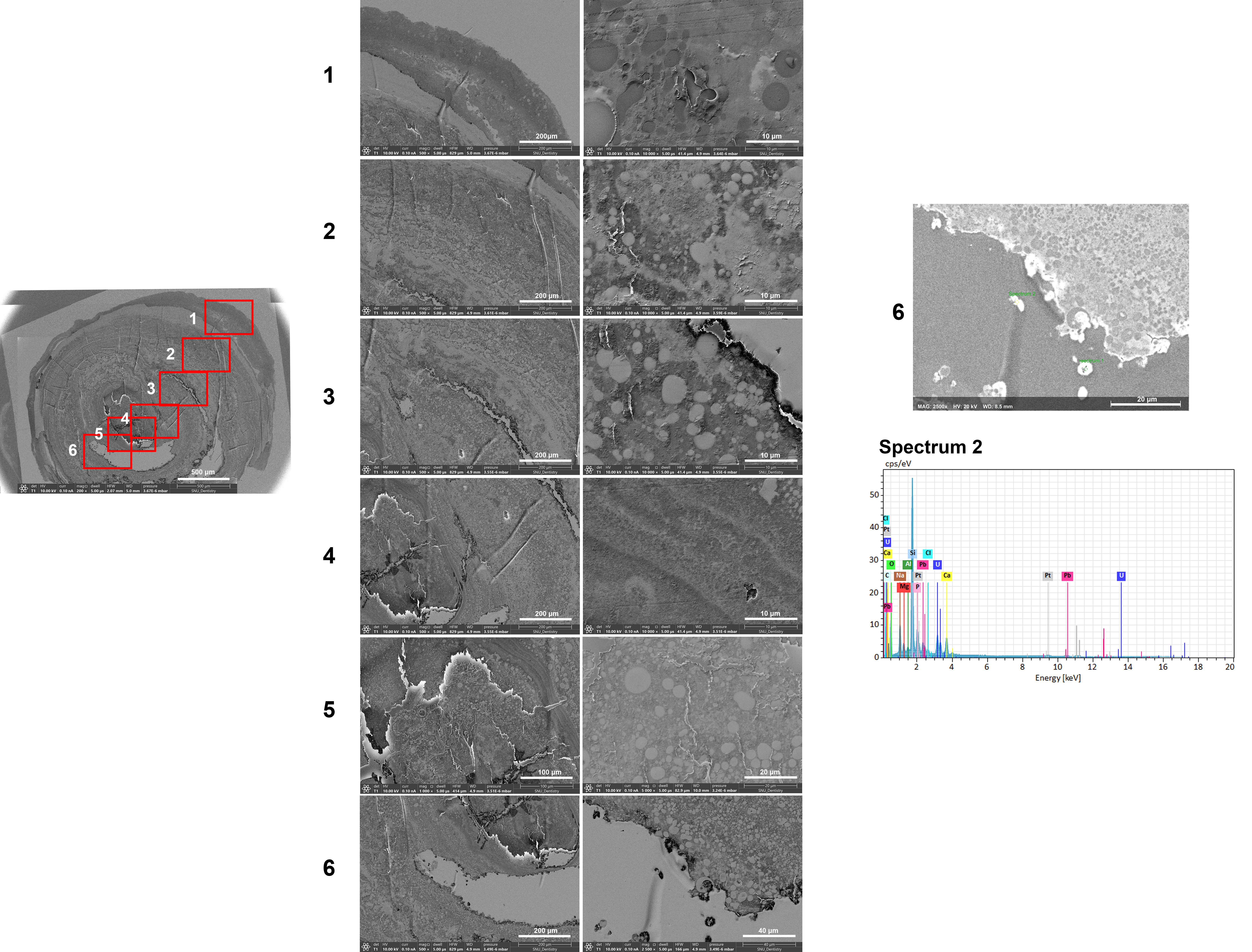

Supplement: Supplementary file 9 — Supplementary Information 9. [file 41598_2023_37462_MOESM9_ESM.jpg]

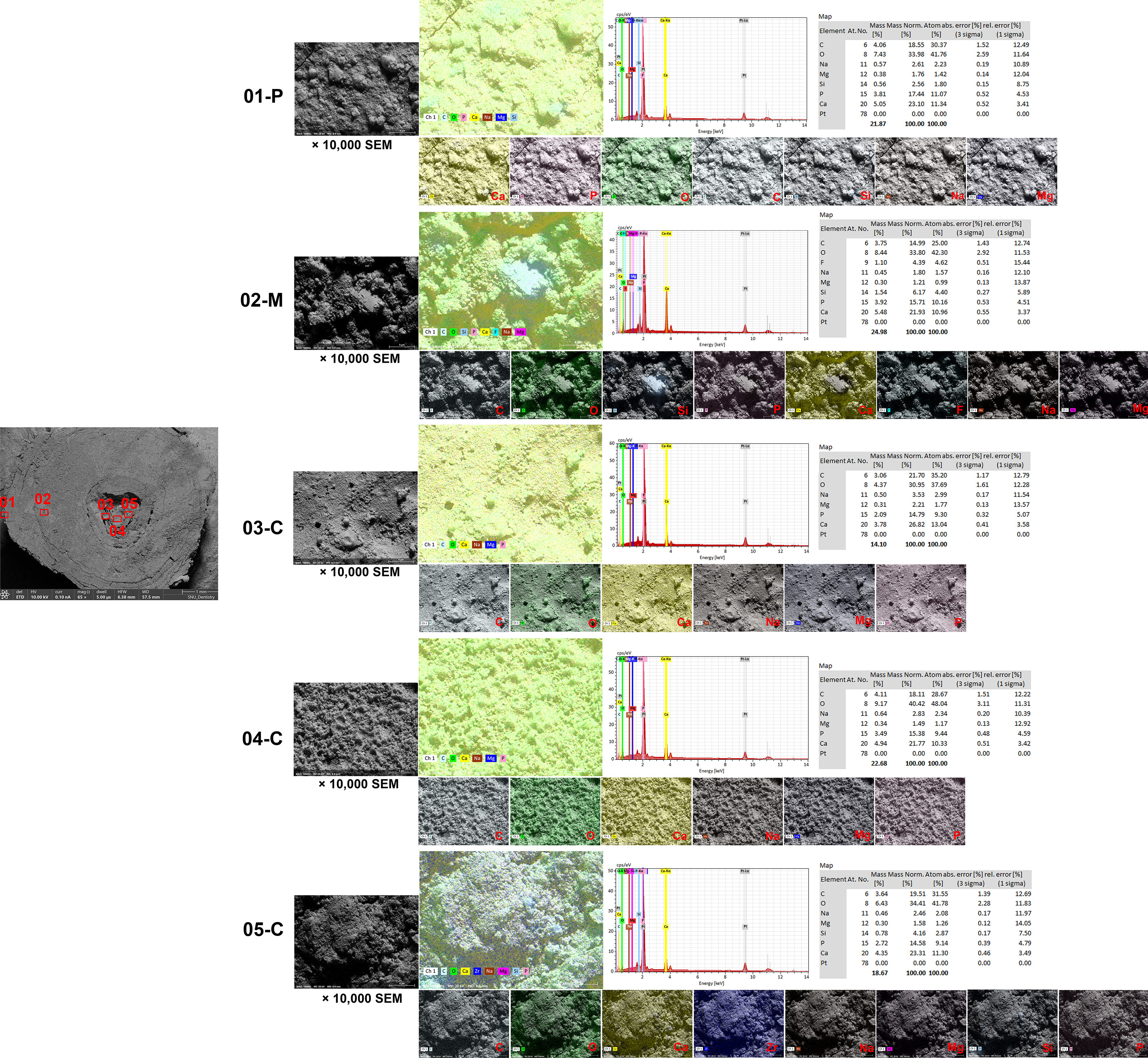

Supplement: Supplementary file 10 — Supplementary Information 10. [file 41598_2023_37462_MOESM10_ESM.jpg]

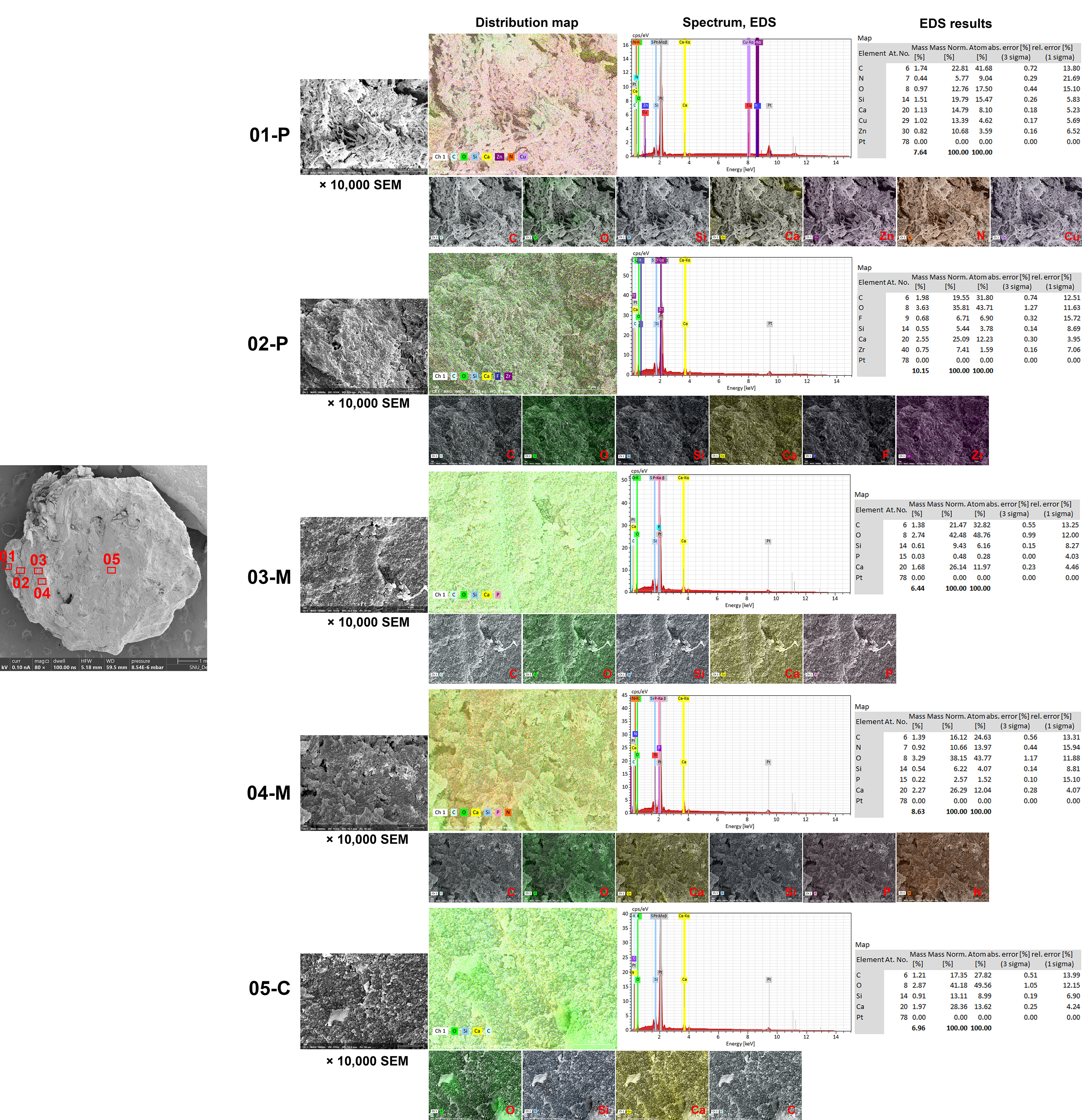

Supplement: Supplementary file 11 — Supplementary Information 11. [file 41598_2023_37462_MOESM11_ESM.jpg]

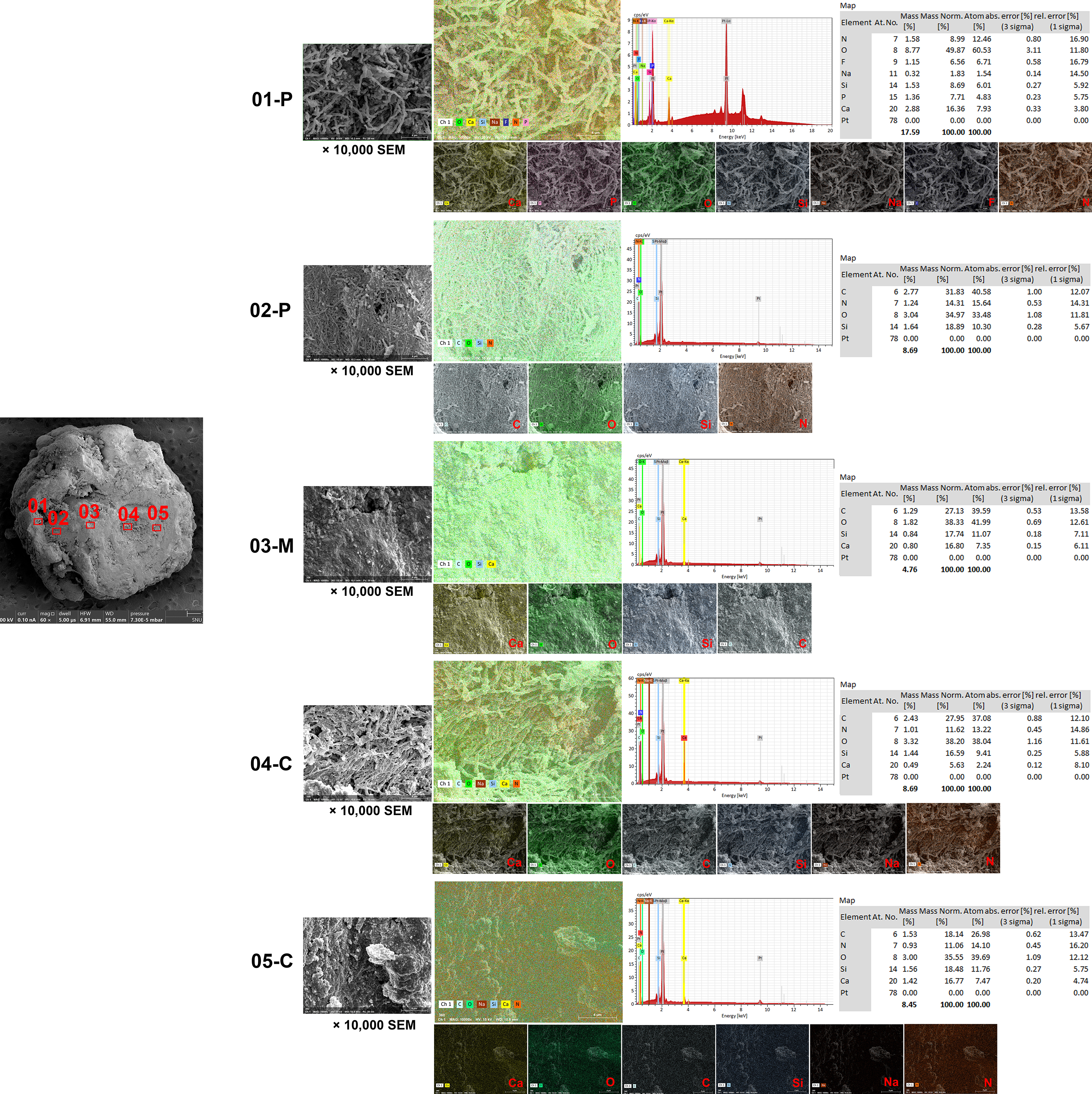

Supplement: Supplementary file 12 — Supplementary Information 12. [file 41598_2023_37462_MOESM12_ESM.jpg]

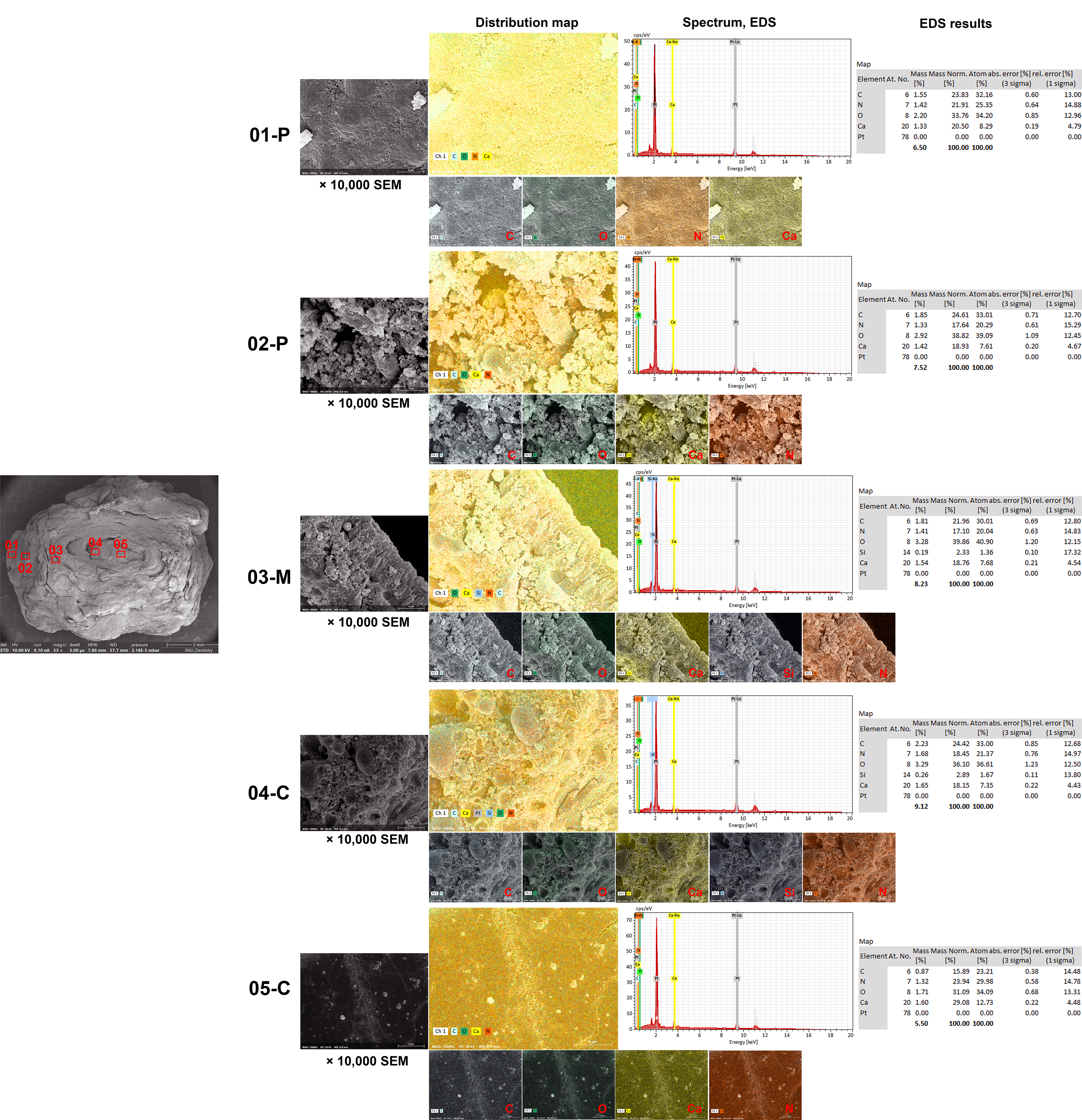

Supplement: Supplementary file 13 — Supplementary Information 13. [file 41598_2023_37462_MOESM13_ESM.jpg]

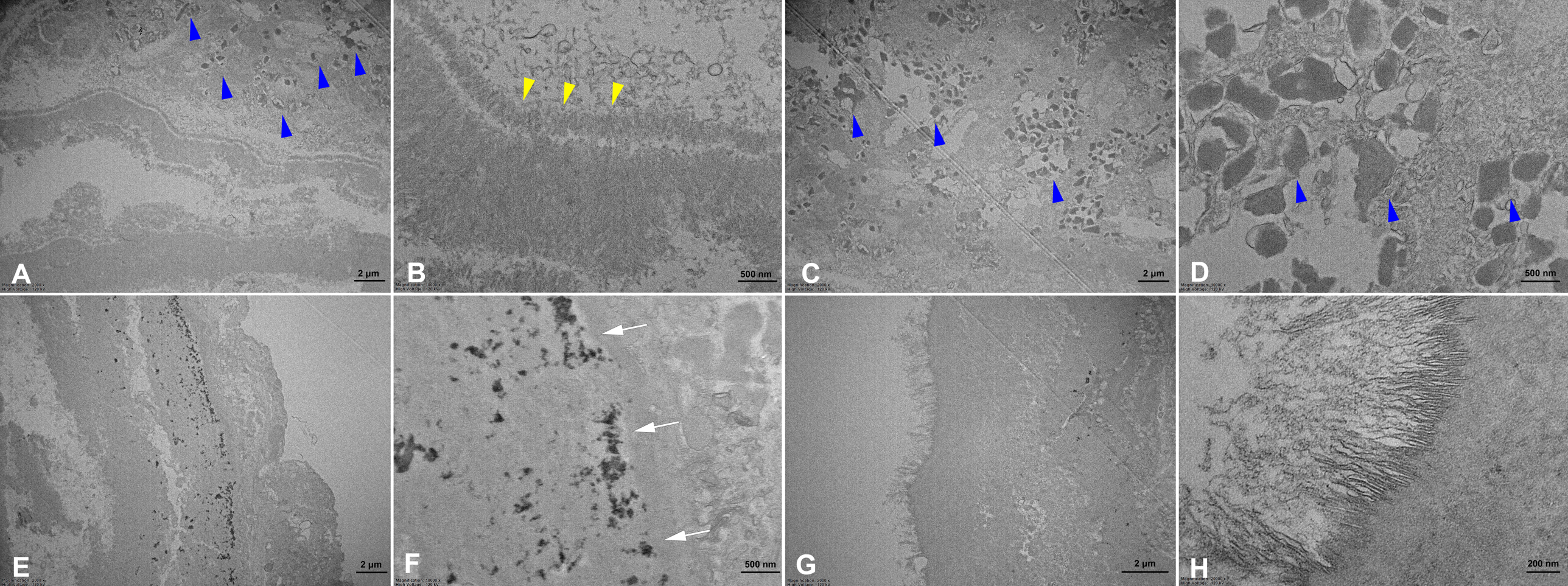

Supplement: Supplementary file 14 — Supplementary Information 14. [file 41598_2023_37462_MOESM14_ESM.jpg]

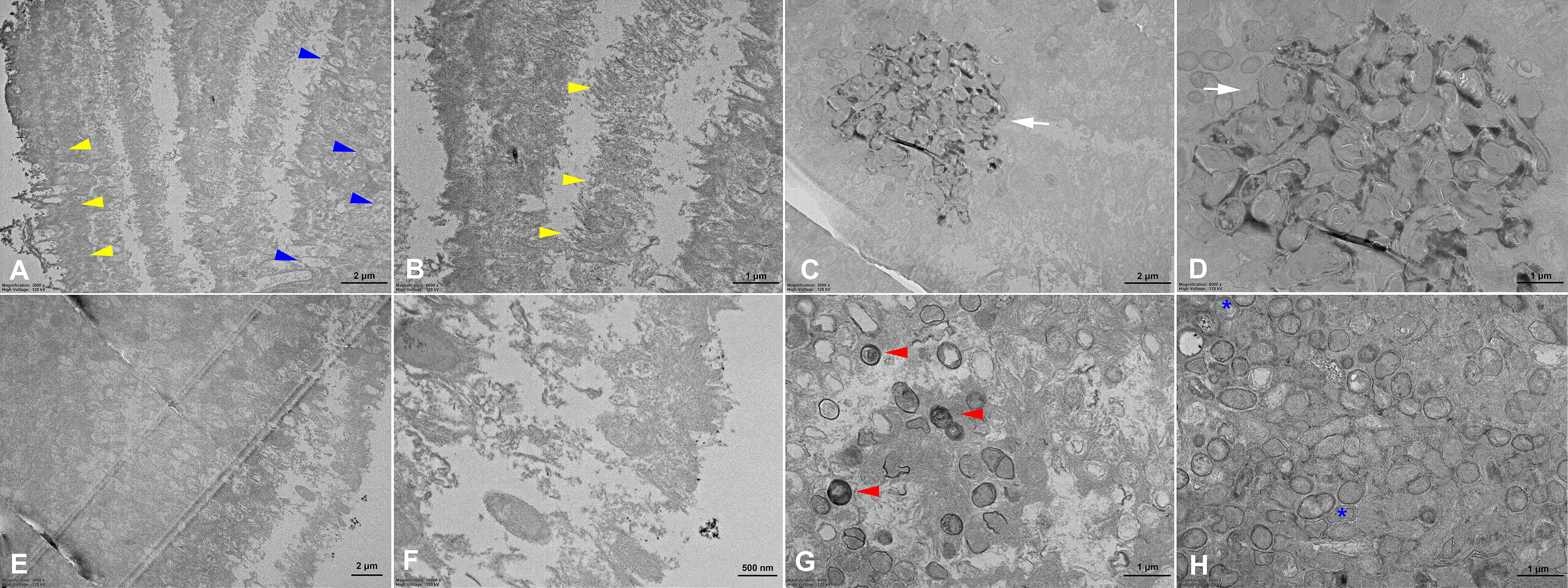

Supplement: Supplementary file 15 — Supplementary Information 15. [file 41598_2023_37462_MOESM15_ESM.jpg]

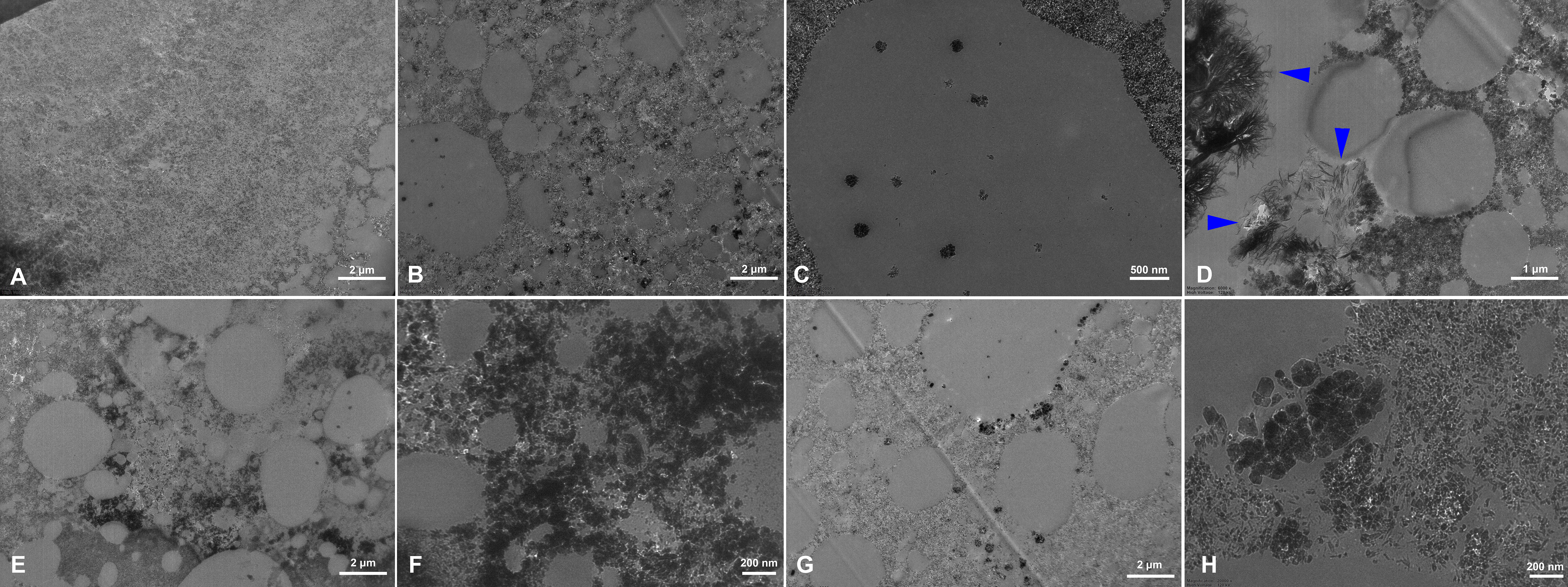

Supplement: Supplementary file 16 — Supplementary Information 16. [file 41598_2023_37462_MOESM16_ESM.jpg]
