# Supplementary material for: Identification of biological components for sialolith formation organized in circular multi-layers
Source: Sci Rep. 2023 Jul 28;13:12277. doi: 10.1038/s41598-023-37462-w (PMC10382579; doi:10.1038/s41598-023-37462-w)
Supplement: Supplementary file 17 — Supplementary Information 17. [file 41598_2023_37462_MOESM17_ESM.pptx]

## Slide 1
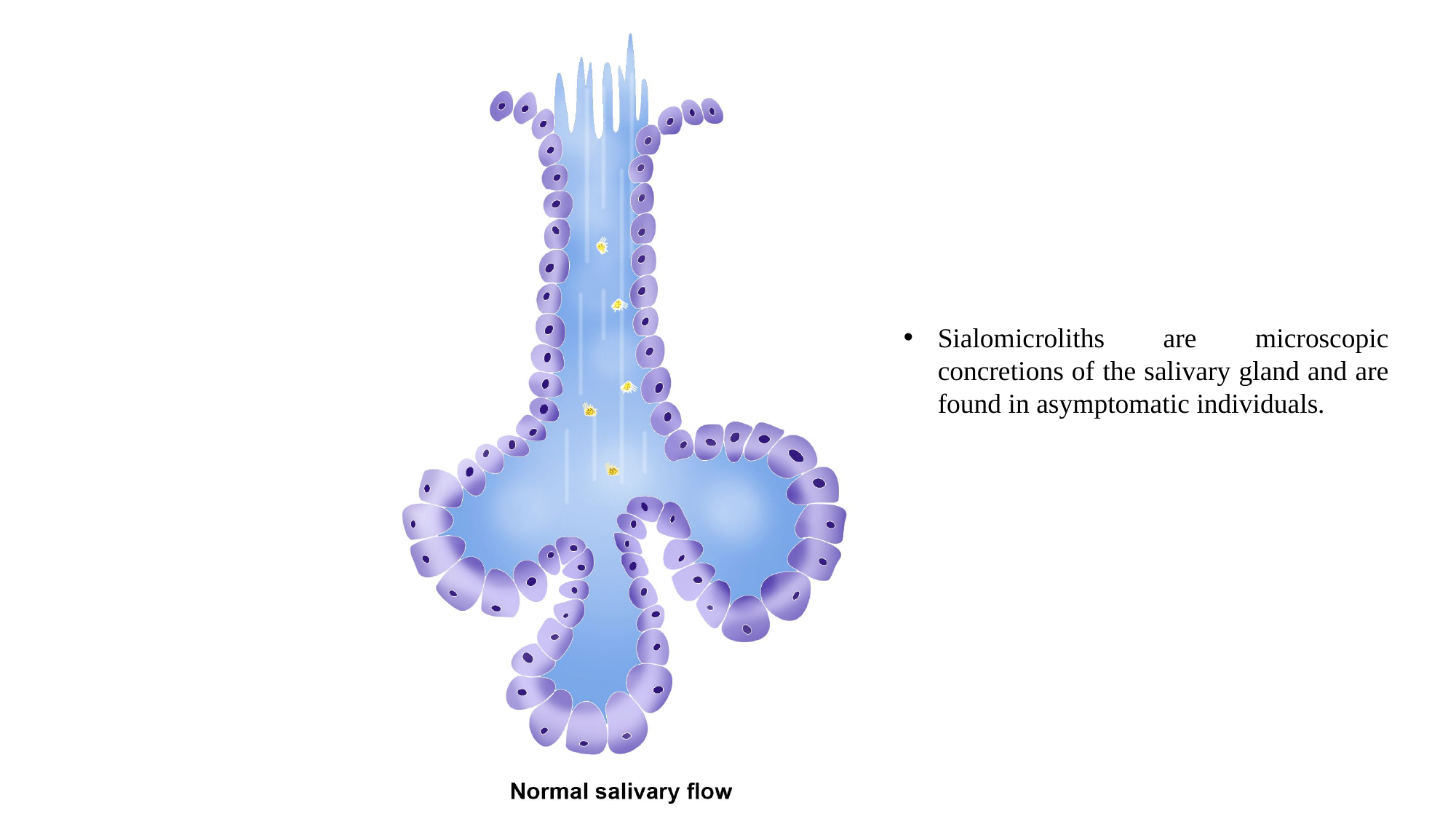

Sialomicroliths are microscopic concretions of the salivary gland and are found in asymptomatic individuals.

## Slide 2
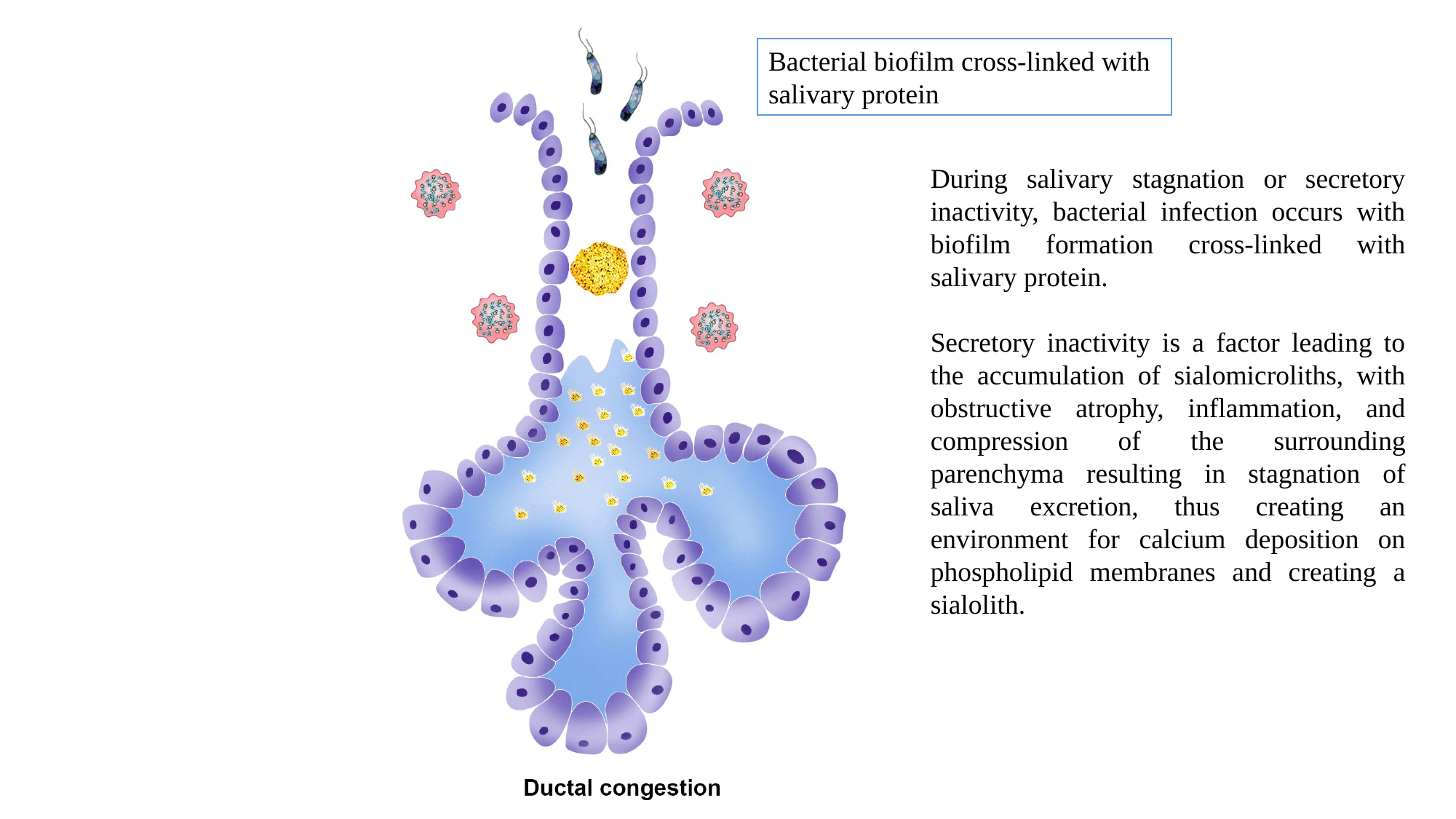

Bacterial biofilm cross-linked with salivary protein
During salivary stagnation or secretory inactivity, bacterial infection occurs with biofilm formation cross-linked with salivary protein.
Secretory inactivity is a factor leading to the accumulation of sialomicroliths, with obstructive atrophy, inflammation, and compression of the surrounding parenchyma resulting in stagnation of saliva excretion, thus creating an environment for calcium deposition on phospholipid membranes and creating a sialolith.

## Slide 3
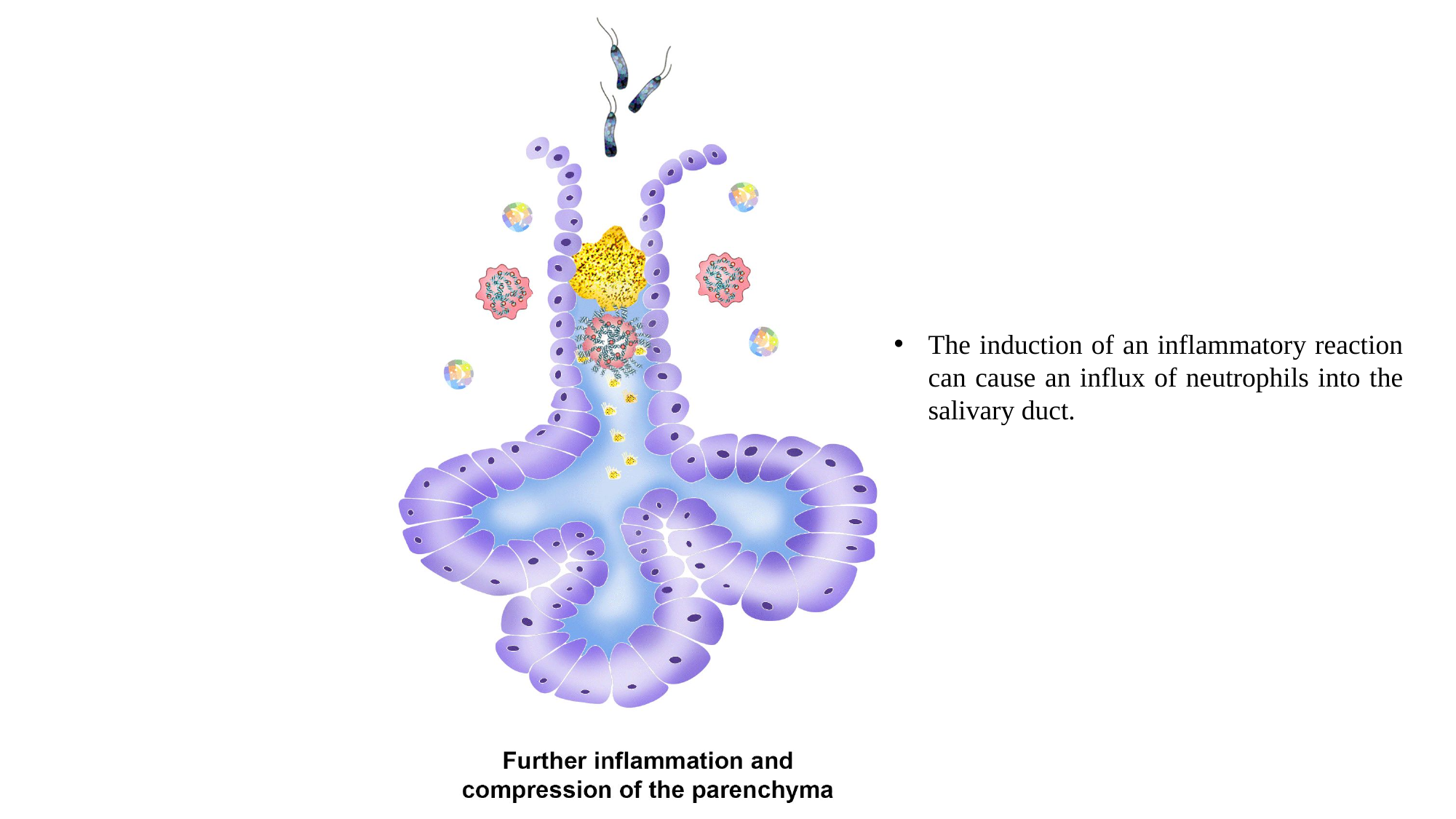

The induction of an inflammatory reaction can cause an influx of neutrophils into the salivary duct.

## Slide 4
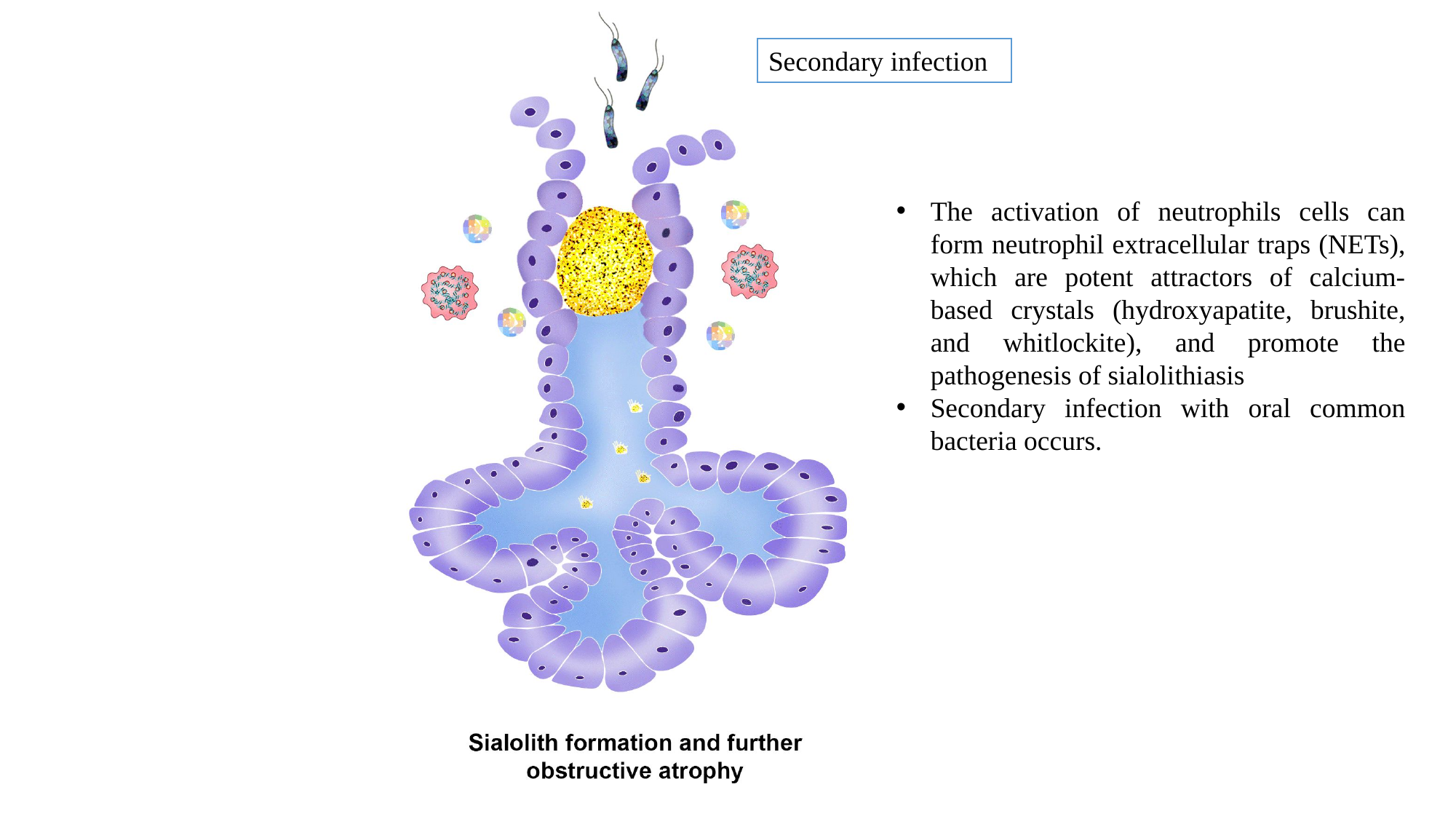

Secondary infection
The activation of neutrophils cells can form neutrophil extracellular traps (NETs), which are potent attractors of calcium-based crystals (hydroxyapatite, brushite, and whitlockite), and promote the pathogenesis of sialolithiasis
Secondary infection with oral common bacteria occurs.
