# Supplementary material for: Identification of biological components for sialolith formation organized in circular multi-layers
Source: Sci Rep. 2023 Jul 28;13:12277. doi: 10.1038/s41598-023-37462-w (PMC10382579; doi:10.1038/s41598-023-37462-w)
Supplement: Supplementary file 18 — Supplementary Information 18. [file 41598_2023_37462_MOESM18_ESM.docx]

**Supplementary figure legends**

**Supplemental figure S3.** Representative histological findings of sialolith found in the hilar portion of the SMG of a 22-year-old female patient (S1) (H&E staining, 4×). Scale bar = 500 μm. (A). Basophilic core (black asterisk), (H&E staining, 20×). Scale bar = 50 μm (B). Mineralized nodules in the outer core (black arrowhead), (H&E staining, 20×). Scale bar = 50 μm (C). Tear-drop-shaped globules (black arrows), (H&E staining, 20×). Scale bar = 50 μm (D). Less mineralized layers of sialolith stained with purple (red arrowhead), nodules undergoing mineralization (blue arrowhead), (H&E staining, 20×). Scale bar = 50 μm (E). Mineralized nodules (black arrowhead), (H&E staining, 20×). Scale bar = 50 μm (F).

**Supplementary Figure S4.** Representative histological findings of sialolith that was found in the hilar portion of the left SMG of a 34-year-old female patient (S3) with two central nidus (black arrow), (H&E staining, 4×). Scale bar = 500 μm. (A). The two central nidus of the sialolith showed poor calcification (black asterisk), (H&E staining, 20×). Scale bar = 50 μm (B-C). Globular structures (black arrowhead) and mineralized nodules were found at the second layer of the sialolith (yellow arrowhead), (H&E staining, 20×). Scale bar = 50 μm (D-F).

**Supplementary Figure S5.** Representative histological findings of a sialolith found in the right Wharton's duct orifice in a pediatric patient (S13) with a single core, (H&E staining, 4×). Scale bar = 500 μm. (A). Eosinophilic core (H&E staining, 20×). Scale bar = 50 μm (B). Laminated structures with concentric patterns and mineralized nodules were observed in the periphery of the sialolith (H&E staining, 20×). Scale bar = 50 μm (C). Amorphous basophilic materials were observed at the middle layer of the sialolith (H&E staining, 20×). Scale bar = 50 μm (D). Bacteria with double membrane (black arrowhead), (H&E staining, 20×). Scale bar = 50 μm (E). Globular structures (black arrow), (H&E staining, 20×). Scale bar = 50 μm (F).

**Supplementary Figure S6.** Combined SEM images at 500× magnification of a sialolith found in the hilar portion of the left SMG in a 24-years old female patient (S5) with 05 focused points of interest. Calcium apatite crystals seen at point 01. A plate-like structure was observed on the point 02. Bacteria were seen on the core nidus area (yellow arrowhead). Calcium apatite structures were seen on points 04 and 05 (Suppl. Fig. S6).

**Supplementary Figure S7.** Combined SEM images at 500× magnification of sialolith found in the hilar portion of right SMG in 50-years-old female patient (S6-2) with 14 points of interest.

**Supplementary Figure S8.** Combined SEM images at 500× magnification of sialolith found in the hilar portion of right SMG in a 48-years-old female patient (S14) with 08 focused points of interest. Alternating layers of organic and inorganic/mineralized bands are observed. Colony of elongated bacteria at the surface of the stone from points 01 and 02 at 20,000× magnification. Calcium apatite crystals from points 03 and 04 at 20,000× magnification. Denser highly mineralized structure is seen on points 03, 04. Bacterial biofilm with single halite-like crystal were seen at the central nidus area at point 05 at 20,000× magnification (blue arrowhead). Dense calcium apatite crystals were seen on points 07 and 08 at 20,000× magnification.

**Supplementary Figure S9**. Backs scattered electron images of a recurrent sialolith found in the hilar portion of left SMG in 47-years-old male patient (S17).

**Supplementary Figure S10**. Mapping of elemental distribution and a spectrum of the representative points with EDS results in sialolith S5. SEM image, 10,000× magnification. EDS analyses were carried out on five representative points of interest on the peripheral (P), middle (M), and core (C) layers. The major elements observed in the specimen were Ca, P, O, and C. A non-homogeneous distribution of Si and Ca were found at 02-M point of interest.

**Supplementary Figure S11**. Mapping of elemental distribution and a spectrum of the representative points with EDS results in sialolith S6-1. SEM image, 10,000× magnification. EDS analyses were carried out on five representative points of interest on the peripheral (P), middle (M), and core (C) layers. A non-homogeneous distribution of O and Ca was found at 01-P point of interest.

**Supplementary Figure S12.** Mapping of elemental distribution and a spectrum of the representative points with EDS results in sialolith S14. SEM image, 10,000× magnification. EDS analyses were carried out on five representative points of interest on the peripheral (P), middle (M), and core (C) layers.

**Supplementary Figure S13.** Mapping of elemental distribution and a spectrum of the representative points with EDS results in sialolith S17. SEM image, 10,000× magnification. EDS analyses were carried out on five representative points of interest on the peripheral (P), middle (M), and core (C) layers.

**Supplementary Figure S14.** Representative TEM images of sialolith from the hilar portion of right SMG in 50-years-old female patient (S6-2). Exosomes (blue arrowheads), cell membrane (yellow arrowheads), magnification 2,000×, 10,000× (A-B). Exosomes, magnification 2,000×, 10,000× (C-D). Deposition of inorganic material (white arrows), magnification 2,000×, 10,000× (E-F). Cell surface of a dead cell, magnification 2,000×, 20,000× (G-H).

**Supplementary Figure S15**. Representative TEM images of pediatric sialolith in a 7-year-old female patient (S13). In the internal lamellas, the globular structure was dominant (blue arrowheads), while the crystalline pattern was heterogeneous in several outer layers, with some regions of needle-like patterns (yellow arrowheads), magnification 2,000×, 6,000×, respectively (A-B). Intracellular components including mitochondria were also found (white arrows), magnification 3,000×, 6,000× (C-D). Intracellular components, magnification 3,000×, 10,000× (E-F). Mitochondria (red arrowheads), magnification 6,000× (G). Endoplasmic reticulum (blue asterisk), magnification 6,000× (H).

**Supplementary Figure S16.** Representative TEM images of recurrent sialolith from the hilar portion of left SMG in 47-year-old male patient (S17). In the peripheral lamella a homogenous layer of organic compounds was found, magnification 3,000× (A). Deposition of large single microcrystalline inorganic compounds were identified, magnification 3,000×, 10,000× (B-C). Needle-like filamentary crystals were arranged in clusters and different directions (blue arrowhead), magnification 6,000× (D). Deposition of inorganic matter in the inner side of the cell, magnification 3,000×, 20,000× (E-F). Clusters of prismatic and hexagonal hydroxyapatite crystals, magnification 3,000×, 20,000× (G-H).
